# Natural Antioxidants in Anemia Treatment

**DOI:** 10.3390/ijms22041883

**Published:** 2021-02-13

**Authors:** Coralia Cotoraci, Alina Ciceu, Alciona Sasu, Anca Hermenean

**Affiliations:** 1 Department of Hematology, Faculty of Medicine, Vasile Goldis Western University of Arad, Rebreanu 86, 310414 Arad, Romania; alcionasasu@gmail.com; 2 “Aurel Ardelean” Institute of Life Sciences, Vasile Godis Western University of Arad, Rebreanu 86, 310414 Arad, Romania; alina_ciceu@yahoo.com (A.C.); anca.hermenean@gmail.com (A.H.); 3 Department of Histology, Faculty of Medicine, Vasile Goldis Western University of Arad, Rebreanu 86, 310414 Arad, Romania

**Keywords:** anemia, iron deficiency, iron overload, hemolytic anemia, β-thalassemia, sickle cell anemia

## Abstract

Anemia, characterized by a decrease of the hemoglobin level in the blood and a reduction in carrying capacity of oxygen, is a major public health problem which affects people of all ages. The methods used to treat anemia are blood transfusion and oral administration of iron-based supplements, but these treatments are associated with a number of side effects, such as nausea, vomiting, constipation, and stomach pain, which limit its long-term use. In addition, oral iron supplements are poorly absorbed in the intestinal tract, due to overexpression of hepcidin, a peptide hormone that plays a central role in iron homeostasis. In this review, we conducted an analysis of the literature on biologically active compounds and plant extracts used in the treatment of various types of anemia. The purpose of this review is to provide up-to-date information on the use of these compounds and plant extracts, in order to explore their therapeutic potential. The advantage of using them is that they are available from natural resources and can be used as main, alternative, or adjuvant therapies in many diseases, such as various types of anemia.

## 1. Introduction

Anemia is defined as a reduction in the number of circulating RBCs [1] or as a condition in which the number of erythrocytes (and subsequently their ability to carry oxygen) is insufficient to meet physiological needs [2]. Anemia is characterized by a decrease of the hemoglobin (Hb) level in the blood (generally less than 13.5 g/dL in men and 12.5 g/dL in women), which results in a reduction in carrying capacity of oxygen. Diseases that decrease Hb production (e.g., iron deficiency, B12, or folate deficiencies) or accelerate its destruction are often the result of a defect in the structure of Hb [3]. Although anemia is frequently diagnosed by a low level of Hb or hematocrit (Htc), it can also be diagnosed by using the number of RBCs, the average mean erythrocyte volume, the number of reticulocytes in the blood, the examination of the blood smear or Hb electrophoresis [4].

Anemia is a major public health problem. It affects people of all ages, especially pregnant women and children. According to statistics, globally, anemia affects 41.8% of pregnant women and 47.4% of preschool children [5]. Moreover, it has negative effects on health and development, including neonatal and perinatal mortality, low birth weight [6], premature birth [7,8], and developmental delays of the children [9]. Clinically, it is characterized by pallor, fatigue, dizziness, difficulty breathing dyspneea, and weakness [10]. In the absence of effective management, anemia can promote decreased cognitive ability, weakened immune system, and increased mortality [11].

Anemia can be classified in terms of pathogenesis and erythrocyte morphology [12]. The pathogenic mechanisms involved in the onset of anemia are inadequate production and loss of erythrocytes as a result of bleeding or hemolysis. Depending on these pathogenic mechanisms, anemia can be divided into hypo-regenerative and regenerative forms. In hypo-regenerative anemia, bone marrow production is low, as a result of impairment of the latter’s function, decreased number of precursor cells, or lack of nutrients [12]. Damage to pluripotent stem cells usually causes pancytopenia (anemia, leukopenia, and thrombocytopenia). All of these can affect normal hematopoiesis or change the microenvironment required for stem cell regeneration, differentiation, and proliferation [13,14]. In the regenerative form, anemia is characterized by an elevated level of erythropoietin in response to decreased Hb and generally reflects a loss of erythrocytes, caused by bleeding or hemolysis. Bone marrow responds appropriately to a low erythrocyte mass by increasing reticulocyte production [12].

Classification of anemias according to erythrocyte morphology is more useful in medical practice [12]. Microcytic anemia is the type of anemia in which circulating RBCs are smaller than usual. The most common cause of this type of anemia is a decrease in the body’s iron reserves, which can have several causes, such as decreased iron in the diet; reduced absorption of iron in the intestine; acute and chronic blood loss; and increased iron demand in certain situations, such as pregnancy and recovery after a major trauma or surgery [15]. The category of microcytic anemias includes iron deficiency anemia (IDA), thalassemia, anemia associated with various chronic conditions (e.g., rheumatoid arthritis, Hodgkin’s lymphoma, chronic infections, and neoplasms), and sideroblastic anemia (e.g., hereditary and intoxication with lead) [12]. Normocytic anemia may be caused by inadequate erythropoietin (EPO) levels or decreased erythropoietin response, reduced erythrocyte survival, and bone marrow infiltration. Most normocytic, normochromic anemias are a consequence of other conditions. A small part of them reflects a primary blood disorder. This may be due to anemia induced by some chronic conditions (inflammation and neoplasia), kidney failure, endocrine failure, bone marrow failure (pure red cell aplasia and aplastic anemia), acute blood loss, and polycystic blood loss [16]. Macrocytic anemia has two forms: megaloblastic (hypersegmented neutrophils) and non-megaloblastic. Megaloblastic anemia is due to impaired DNA synthesis induced by folate and/or vitamin B12 deficiency [17,18].

The main function of RBCs in the blood is the transport of respiratory gases (oxygen (O_2_) and carbon dioxide (CO_2_)) to and from tissues, by binding gases to Hb inside erythrocytes. Hb is a tetrameric protein consisting of two α- and two β-polypeptide chains. Each of them contains an iron heme group capable of binding an oxygen molecule [19]. Each heme group contains an (Fe^2+^) atom, which binds an oxygen molecule. Thus, a Hb tetramer can bind four oxygen molecules [20].

In the human body, iron is mainly found in erythrocytes, in the form of Hb (about 2 g of iron in men and 1.5 g in women) and, to a lesser extent, in storage compounds (ferritin and hemosiderin), and in muscle cells like myoglobin. Iron is also found bound to proteins (hemoproteins) and non-heme enzymes involved in oxidation-reduction reactions and electron transfer (cytochrome and catalase) [21,22,23].

About 2.2% of the total amount of iron in the body is found in a labile reserve, which forms reactive oxygen species through the Fenton reaction that forms complexes with a class of drugs known as chelators. Iron chelators are used in the treatment of iron overload, a condition often caused by blood transfusions that are used to treat thalassemia and other types of anemia [24,25].

Upon exposure to oxygen, iron forms insoluble oxides that cannot be absorbed into the human gastrointestinal tract. Human enterocytes contain membrane-bound apical enzymes that have the ability to reduce insoluble iron (Fe^3+^) to an absorbable (Fe^2+^) form. Iron overload can be particularly harmful to the heart, liver, and endocrine organs. Excess ferrous iron forms hydroxyl free radicals through the Fenton reaction, which causes tissue damage through oxidative reactions with lipids, proteins, and nucleic acids. Thus, the absorption of iron from the diet and the factors that affect the bioavailability in the body are strictly regulated [26].

The most effective method used to treat anemia is blood transfusion [27]. Oral administration of iron-based supplements is an effective and, at the same time, inexpensive method used to treat patients with iron deficiency anemia. Moreover, this method of treatment is associated with a number of side effects, such as nausea, vomiting, constipation, and stomach pain, which limits its long-term use. In addition, oral iron supplements are poorly absorbed in the intestinal tract, due to overexpression of hepcidin, a peptide hormone that plays a central role in iron homeostasis [28]. If iron supplements are not effectively assimilated by the body, or if their absorption is inhibited, parenteral administration is recommended. Long-term parenteral administration of iron supplements can lead to hyperpigmentation of the skin. Moreover, one of the side effects of taking iron-based supplements is increased free radical production [27]. The use of inappropriate doses of iron-based supplements can induce oxidative stress [29,30], with the formation of oxidation products, and can lead to cardiovascular, neurological, or cancer conditions [31,32,33]. The presence of an excess of free iron initiates the Fenton reaction, which leads to oxidative damage to cell membranes, proteins, lipids, and nucleic acids, as well as the stimulation of inflammatory mediators [34,35].

Therefore, in the case of patients with anemia, the aim is to reduce the dose of iron and replace it with other complementary treatments.

The therapeutic use of herbal products in common clinical practice is not yet regulated for several reasons, such as the lack of efficacy and toxicity data that are required for their approval by health authorities. To these are added the competition of large pharmaceutical companies that make remarkable profits from the sale of synthetic drugs [36]. Currently, there is an increase in the number of clinical trials with herbal therapeutic products used in various diseases, in order to confirm their therapeutic value and receive the necessary approvals for their marketing [37]. Some phytochemicals or herbs act directly to induce the resolution of anemia, and others act pleiotropically through their antioxidant activity, by increasing oxidative stress resistance or by triggering cellular mechanisms, such as autophagy [38], or, for example, by targeting inflammation in the elderly population and subsequently reducing the anemia associated with chronic inflammation [39].

In this review, we conducted an analysis of the literature on biologically active compounds and plant extracts used in the treatment of various types of anemia. The purpose of this review is to provide up-to-date information on the use of these compounds and plant extracts, in order to explore their therapeutic potential. The advantage of using them is that they are available from natural resources and can be used as main, alternative, or adjuvant therapies in many diseases, such as various types of anemia.

## 2. Natural Antioxidants in Iron Deficiency Treatment

Iron is essential for the production of Hb. Depletion of iron deposits can be caused by blood loss, decreased iron intake, impaired absorption, or increased demand. Iron deficiency anemia can be caused by occult gastrointestinal bleeding [40] or decreased iron in the diet, or reduced iron absorption [26], accounting for more than half of all types of anemia [11] and require iron supplementation.

Due to the fact that oral iron supplements are associated with side effects, such as gastrointestinal irritations, reduced bioavailability, and lipid peroxidation [41], it is necessary to develop new iron-based supplements without or with minimal side effects. Polysaccharide chelated iron is characterized by high stability, water solubility, and fewer side effects [42]. Other polysaccharide–iron complexes have been used in the treatment of IDA, such as iron–dextran, iron–starch, and Niferex (a combination of ascorbic acid and iron–polysaccharide complex) [43,44].

*Ulva prolifera* is one of the most widespread species of green macroalgae [45]. The sulfate polysaccharides from *Ulva prolifera* (SUE) are a group of sulfated heteropolysaccharides with unique structural features in the form of rhamnose and uronic acid linkages of (1 → 4) -linked β-L-arabinopyranose residues [46]. In the case of rat-induced IDA, the SUE-iron (III) complex induced an increase in the number of RBCs and serum iron levels and contributed to the restoration of normal Hb levels [47].

*Angelica sinensis* has been shown to improve hematopoiesis by increasing the secretion of hematopoietic growth factors, such as erythropoietin, by stimulating hematopoietic cells and muscle tissue [48]. Polysaccharides from *A. sinensis* (ASP) improved serum iron levels and participated in the regulation of iron homeostasis [49]. Moreover, Liu et al. (2012) obtained ASP from the root powder of *A. sinensis* riched in arabinose, glucose, and galactose, with a molar ratio of 1: 5.68: 3.91 [50]. ASP has been shown to decrease hepcidin expression by inhibiting SMAD_4_ expression in the liver and stimulating erythropoietin secretion, while other results showed that the decreased hepatic hepcidin expression is due to inhibition of the expression of JAK1/2, phospho-JAK1/2, phospho-SMAD1/5/8, phospho-ERK1/2, and stimulating SMAD7 [51]. Previously, ASPs have been shown to activate erythropoiesis [52,53].

Beetroot (*Beta vulgaris*) contains iron, nitrates, sodium, potassium, and betalaine [54]. Among the benefits of beetroot juice are the treatment of anemia by improving the ability of erythrocytes to carry oxygen, lowering blood pressure by dilating blood vessels and relaxing smooth muscles, preventing birth defects by increasing folate levels, etc. [55]. Compared to other vegetables with a high iron content, beets have a low price and are easy to store [56]. Consumption of 8 g of beets for 20 days induced an increase in Hb, ferritin, and serum iron levels, as well as a decrease in transferrin and total iron-binding capacity levels in seven women aged 22–24 years [57]. Consumption of beetroot in the form of juice (100–200 mL) increased the level of Hb [58,59]. Moreover, administration of 200 mL of beet juice for six weeks induced the increase of HTC, RBC, iron, and ferritin levels [58]. The administration of beetroot in the form of powder and iron-based supplements for 14 days in women with anemia led to increased levels of Hb, HTC, and erythrocyte counts [60].

The results of the in vitro and in vivo studies regarding pharmacological effects exerted by the natural antioxidants in iron deficiency anemia are summarized in Table 1.

## 3. Natural Antioxidants in Iron Overload Treatment

Iron overload is associated with aplastic anemia, sideroblastic anemia, Blackfan-Diamond anemia, Fanconis anemia, pernicious anemia, congenital dyserythropoietic anemia, hereditary hypochromic anemia, or haemoglobinopathies, as β-thalassaemias or sickle cell anaemia.

Although blood transfusions are important for patients with anemia, chronic transfusions inevitably lead to iron overload, as the body cannot eliminate excess iron. If not treated properly, the cumulative effects of iron overload led to morbidity and mortality [73]. One unit of transfused RBCs contains about 250 mg of iron [74], while the body cannot excrete more than 1 mg of iron per day. A patient who receives 25 units per year accumulates 5 g of iron per year in the absence of chelation [75].

Chelation therapy is used in binding the iron excess and removing it from the body. Synthetic iron chelators are toxic in high doses. Due to the high costs, toxicity and side effects of treatment with synthetic iron chelators, a large number of patients are currently not receiving any iron chelation treatment. There are a number of orally active antioxidants that have the ability to chelate iron and eliminate free radicals. They also have a lower cost and toxicity compared to synthetic drugs. These natural chelators form complexes with metals and could be used in the treatment of iron overload without inducing another micromineral deficiency, being an advantage that synthetic drugs do not have [76].

The natural iron chelators contain a catechol or gallate fragment that acts as a binding site for metals and further is eliminated from the body. In addition to the ability to chelate iron and eliminate free radicals, these antioxidants can be effective in iron overload by reducing the iron load in the liver, increasing iron excretion in feces and urine, reducing serum ferritin, removing iron from ferritin and transferrin, increasing hepcidin expression, reducing iron absorption in the intestine, increasing iron absorption and incorporation into the heme, and inducting osteo- and cardio-protective effects [76].

Flavonoids and polyphenolic compounds with at least two iron binding sites have iron chelating properties. These flavonoids fall into two categories: lipophilic and hydrophilic chelators. Lipophilic chelators increase iron absorption, reduce iron excretion, and increase the deposition of excess iron in tissues. Therefore, they are a possible treatment for iron-deficiency anemia. Hydrophilic chelators, on the other hand, favor the elimination of excess iron, reduce iron absorption, and exert antioxidant and anti-inflammatory activity, without having other side effects [36]. Combining synthetic iron chelators with these antioxidants, or even replacing them with chelators from natural sources, would be a possible treatment for iron overload [76]. By chelating iron, flavonoids decrease high oxygen toxicity, for example, by inhibiting HO • production from the Fenton reaction [77]. The mechanism by which certain flavonoids reduce the bioavailability of non-hemic iron is not fully understood, but it is assumed that flavonoids have the ability to chelate non-hemic iron [78,79,80,81,82].

Grape seed extract (GSE) contains various polyphenolic compounds, such as gallic acid, catechin, epigallocatechin-3-gallate (EGCG), epigallocatechin, epicatechin-3-gallate, epicatechin, and proanthocyanidins [83]. GSE extracts rich in polyphenols have antioxidant activity due to the phenolic compounds ability to neutralize free radicals and to chelate certain metals, such as iron [84,85]. Moreover, it was found that EGCG and GSE increased absorbtion on hemic iron on apical side and blocked transepithelial transport [83] or inhibited intestinal absorption of nonhemic iron [86].

Curcumin, the main curcuminoid in *Curcuma longa* L. (turmeric), is a low-molecular-weight polyphenol, widely used in Ayurveda and Chinese medicine [87]. Turmeric type 97 (77% curcumin, 17% dexethoxycurcumin, and 3% bisdemethoxy curcumin) induced an increase in the level of transferrin receptor 1 (TfR1) and the induction of activated iron-regulatory proteins (IRPs), a decrease of the hepatic ferritin level and its H and L subunits [88]. Other results show that 1000 mg iron/kg body weight and curcumin increased TfR1 and iron-responsive element-binding protein (IRP), favored the appearance of hypochromic RBCs, and decreased the levels of Hb, HTC, serum iron, ferritin, hepcidin, and transferrin saturation, as well as iron levels in the spleen and bone marrow [89].

Quercetin is a flavonol found in onions, broccoli, garlic, tomatoes, black tea, spinach, and apples [90,91,92], recognized for their antioxidant and anti-inflammatory activity [93]. Quercetin increased the expression of hepcidin, one of the main hormones involved in intestinal absorption of iron, which could involve the Nrf2 pathway [94]. Granado-Serrano et al. (2012) demonstrated that quercetin can activate the Nrf2 pathway by supporting nuclear translocation and its transcriptional activity [95]. Given that the levels of ferroportin (FPN) and ferritin are overexpressed transcriptionally by the Nrf2 pathway, quercetin could affect iron homeostasis and help cells to fight against oxidative stress [96]. Prenatal exposure of mice to quercetin resulted in increased hepatic iron deposits and induced overexpression of hepcidin to adults [77].

Quercetin chelates metal ions in a stable complex, thus preventing the Fenton reaction [97], and protects human erythrocytes from iron-induced oxidative damage [98,99]. Quercetin can also act as a siderophore through glucose transporters [100]. Similar to other flavonoids, it is thought to form a complex with Fe^3+^ that has greater stability than Fe^2+^. Even if quercetin initially forms a complex with Fe^2+^, it will be further self-oxidized, resulting in Fe^3+^ [101]. Iron chelation by the 3-hydroxyl group of quercetin is an important factor in iron absorption in the duodenum [79]. The decrease in duodenal iron transfer is due to the chelation of iron by quercetin, which increases the apical absorption of iron, but prevents basolateral transport. However, the precise site of iron chelation by quercetin is unknown. It is not known whether chelation occurs in the duodenal lumen or in the cytosol of duodenal enterocytes [96].

Myricetin (3,5,7,3′,4′,5′-hexahydroxyflavonol) is a flavonoid initially isolated from the bark of the *Myrica rubra* that has been shown to have iron-chelating properties [80,102]. Administration of myricetin to C57BL/6 mice favored an increase in RBCs, Hb levels, and serum iron and decrease in the hepatic expression of hepcidin and splenic iron levels [103].

Silibin is a biologically active compound from silymarin [104], a flavonolignan raported to have iron chelating effect [105], while other studies reported a high affinity for Fe (III) iron-silibin complex in acidic pH [104,106]. Bares et al. (2008) observed that oral administration of silibin for 12 weeks reduced iron deposits in patients with chronic hepatitis C [107].

The main pharmacological effects of the natural antioxidants in iron chelation activities are summarised in Table 2.

## 4. Natural Antioxidants in the Treatment of Hemolytic Anemia

Hemolytic anemia is a normocytic anemia characterized by low Hb levels due to the destruction of RBCs and increased hemoglobin catabolism. Depending on where the hemolysis occurs, it can be intravascular or extravascular [126]. Destruction can also occur in the case of inherited protein deficiencies (membranopathies, i.e., hereditary spherocytosis), fragmentation (i.e., microangiopathic hemolytic anemia, thrombotic thrombocytopenic purpura, and disseminated intravascular coagulation), antibodies that bind to RBC resulting in phagocytosis (immune-mediated), drug-induced hemolysis, infections, or direct trauma [127].

The results of the in vitro and in vivo studies regarding pharmacological effects exerted by the natural antioxidants in hemolytic anemia are summarized in Table 3.

## 5. Natural Antioxidants in the Treatment of Thalassemia

Thalassemias are a group of inherited diseases that lead to a defective hemoglobin production. Patients with thalassemia have a mutation that affects the production of the hemoglobin globin polypeptide chain and is associated with inefficient erythropoiesis. It is characterized by decreased HbA production secondary to low beta-globin chain production and stopping maturation due to apoptosis of erythroid precursors induced by excess alpha chain precipitates [150].

Iron overload is a common complication of thalassemia syndromes, which can lead to organ damage and increased mortality [151,152]. Iron-induced toxicity in β-thalassemia is the leading cause of oxidative stress. Oxidative stress, associated with the formation of reactive oxygen species (ROS), plays an important role in the development of inflammation, decreased plasma antioxidant levels, depletion of erythrocyte glutathione (GSH), increased lipid peroxidation of RBCs membranes and immunosuppression in the affected patients [153,154]. Moreover, iron overload in patients with β-thalassemia decreases T cell proliferative activity [155,156].

Flavonoids and phenolic compounds have antioxidant properties, ability to neutralize free radicals [157,158,159,160] and metal chelation, suggesting that they may have a protective effect under oxidative stress-based pathological conditions caused by iron overload [161]. Therefore, the use of polyphenols as iron chelators has been proposed in clinical practice [162].

Silymarin, isolated from *Silybum marianum*, is a powerful antioxidant and has hepatoprotective and iron chelating activities [163], being introduced as an adjuvant without side effects in numerous clinical trials [164]. Gharagozloo et al. (2013) recommended the use of silymarin as a possible herbal immunomodulatory drug in the treatment of patients with β-thalassemia due to its antioxidant, cytoprotective and iron chelating activity. The study included 59 β-thalassemia patients who received 140 mg of silymarin and desferioxamine (DFO) three times daily for 3 months. Combination therapy has been well tolerated and more effective in reducing serum ferritin levels than administering DFO alone, in increasing GSH level of RBCs and promoting a decrease in serum iron and ferritin [165]. In another clinical study, patients were treated with a combination of DFO and silymarin (420 mg/day) or DFO for 9 months. Silymarin treatment significantly reduced serum iron, ferritin, serum hepcidin, and soluble transferrin receptor (sTfR), demonstrating the beneficial potential of silymarin as an iron chelating agent in reducing the serum ferritin and iron level in β-thalassemia [166]. Similar results were obtained by Hagag et al. (2015) in a clinical study for silymarin in combination with deferiprone (DFP) [167], as well by the combination of deferasirox (DFX) and silymarin [168]. Serum iron levels decreased significantly [168]. Therefore, the effects of iron chelating silymarin are related to its Fe (III) binding capacity.

The results of the studies regarding the pharmacological activities exerted by natural in β-thalassemia treatment are summarized in Table 4.

Beta-thalassemia major is a hereditary haemolytic anemia in the treatment of which multiple blood transfusions are used [177]. Patients with major thalassemia, also known as Cooley’s anemia, have severe and hypochromic microcytic anemia, associated with an increased number of RBCs and a low level of mean corpuscular volume (MCV) and mean corpuscular Hb (MCH). Peripheral blood smear highlights microcytosis and hypochromia, anisocytosis, poikilocytosis, and nucleated RBCs (e.g., erythroblasts). The number of erythroblasts correlates with the degree of anemia and is significantly increased after splenectomy [178].

Finding natural iron chelators of plant origin that also act as a hepcidin agonist may be useful in the management of excess iron in patients with β-thalassemia major [179].

The results of the studies regarding the pharmacological activities exerted by natural in β-thalassemia major are summarized in Table 5.

## 6. Natural Antioxidants in the Treatment of Sickle Cell Anemia

Sickle cell anemia is inherited as an autosomal recessive condition [185]. Sickle cell disease is one of the most notable impairments in the structure of hemoglobin. While the amount of hemoglobin produced may be normal, the substitution of the amino acid valine with glutamic acid results in a structural defect that favors the polymerization of deoxygenated hemoglobin [186]. It is characterized by the presence of sickle-shaped cells that block blood flow through the spleen, causing splenic sequestration [185]. When deoxyhemoglobin polymerizes, it forms fibers that alter the shape of erythrocytes [186]. Repeated stress caused by sickle cell disease will damage circulating erythrocyte membranes, leading to premature cell death. While sickle cell anemia may remain asymptomatic for a significant period of time, severe hypoxia may cause a seizure, with symptoms of generalized pain, fatigue, headache, jaundice, and repeated vascular occlusion (stroke, etc.) [3].

Given the increasing mortality rate of patients with sickle cell disease, especially in children, and the side effects of chemotherapy, the addition of natural products (phytomedicines/herbal drugs) in the treatment is beneficial [187]. Several herbal extracts have properties in sickle cell anemia treatment, but there is still no promising drug on the market for the treatment of this condition [188,189,190,191]. The active constituents of medicinal plants and natural compounds, known as antisickling agents, are rich in aromatic amino acids, phenolic compounds, and antioxidants [192] and acts as antioxidant therapy to ameliorate the complications of sickle cell anemia [193]. Antioxidants have many beneficial effects, protect against RBC lipid peroxidation, increased glutathione levels (GSH), and reduce ROS levels [194].

Rutin (quercetin-3-rhamnosyl glucoside) is a flavone intentive studied for its antioxidant properties [195,196,197]. Rutin has antiplatelet and protective effects of the vascular endothelium against oxidative stress in sickle cell anemia [198,199]. Moreover, it restored the integrity of the erythrocyte membrane, prevented and reversed lipid peroxidation, induced increased GSH and CAT levels, and decreased SOD activity. The beneficial effects of rutin in sickle cell anemia may be associated with modulation of deoxy-hemoglobin and alteration of redox homeostasis. Similar results were obtained fot chrysin [199].

The main pharmacological effects of the phytochemicals/herbs in sickle cell anemia treatment are summarized in Table 6.

## 7. Natural Antioxidants in the Treatment of Aplastic Anemia

Aplastic anemia is a condition in which the bone marrow is destroyed and blood cell production is diminished [231]. This usually correlates with a deficiency of erythrocytes (anemia), leukocytes (leukopenia), and platelets (thrombocytopenia) [232,233]. Aplastic anemia refers to the syndrome of chronic primary hematopoietic insufficiency due to lesions, leading to diminution or absence of hematopoietic precursors in the bone marrow [234,235].

Aplastic anemia can be caused either by extrinsic suppression mediated by hematopoietic stem cell immunity or by intrinsic bone marrow progenitor abnormality [236,237]. Damaged hematopoietic stem cells mature into self-reactive T-helper (T1) cells that release cytokines: interferon (IFN) and tumor necrosis factor (TNF) to develop a cytotoxic cascade to kill and suppress other hematopoietic stem cells. The exact antigens of T1 target cells are unclear, but one appears to be one of the glucose inositol phosphate-bound (GPI) proteins on cell membranes. Moreover, genes involved in apoptosis are overexpressed [238].

Strategies recently applied in the treatment of aplastic anemia include immunosuppression and/or hematopoietic stem cell transplantation [239,240]. Numerous lymphocytotoxic agents have been widely used, but some of them have various adverse effects, such as anaphylaxis fever, chest pain, and diarrhea [241].

In recent years, natural herbal products have attracted much attention, being used as an effective and safe alternative treatment for bone marrow failure [242].

For example, saponins extracted from *Panax notoginseng* (PNS) induced the proliferation of hematopoietic stem/progenitor cells and stromal cells in vitro [243,244,245,246,247], probably by overexpressing genes involved in hematopoiesis, such as GR-NTF [243]. PNS activated the MAPK pathway and GATA transcription factors in hematopoietic cells [245]. Moreover, it has been shown to differentiate the mesenchymal stem cells and NIH3T3 cells [244,246].

The main pharmacological effects of the phytochemicals/herbs in anaplastic anemia treatment are summarized in Table 7.

## Figures and Tables

**Table 1:** Pharmacological effects of natural antioxidants in iron deficiency anemia.

Bioactive Compound	Doses	In Vitro/ In Vivo/ Clinical Study	Model	Bioactive Effect	References
Iron from *Moringa leaves*	10% and 20% dehydrated *Moringa leaves*	in vivo	iron-deficient diet in Wistar rats	↑ serum iron, ferritin, and transferrin concentration Dietary iron from *Moringa leaf* was found to be superior compared with ferric citrate (35 mg/kg) in overcoming the effects of iron deficiency in rats	[61]
Sulfate *Ulva* polysaccharide (SUE)	Low-dose group (SUE-iron (III) with iron concentration of 0.8 mg/mL, 0.7 mg kg^−1^ BW, Fe) High-dose group (SUE-iron (III) with iron concentration of 2.3 mg/mL, 2.0 mg kg^−1^ BW, Fe)	in vivo	iron deficiency anemia in Wistar rats	↑ RBC number ↑ serum iron ↓ TIBC ↓ IL-4 returnes Hb to the normal levels	[47]
*Astragalus membranaceus* polysaccharide-iron (III) complex	APS-iron (III) complex with the iron content 12.5, 25, 50, 75, and 100 mg/kg	in vivo	Iron-deficiency anemia rodent model	↑ Hb ↑ SOD and CAT ↓ MDA	[62]
Ginger + iron supplem.	215 mg	clinical study	iron deficiency anemia 62 pacients 18–65 years	↑ plasma iron ↑ plasma ferritin ↓ TIBC	[63]
Quercetin + FeSO_4_	50 mg·kg^−1^ quercetin + 50 mg·kg^−1^ FeSO_4_	in vivo	Iron deficiency anemia rat model	↑ Hb ↑ serum iron level ↑ iron stores in spleen ↑ expression of SLC40 -improve red blood cells level -↓ CD68 macrophage activation -↓ iNOS expression in splenic red pulp	[64]
Low-molecular-weight polysaccharide from *Enteromorpha prolifera* (LPE)-iron (III) complex	Low LPE-iron (III) complex group (0.7 mg Fe/kg body weight) High LPE-iron (III) complex group (2 mg Fe/kg body weight)	in vivo	iron deficiency anemia rat model	- improved the growth of rats with IDA ↑ Hb ↑ RBC numbers ↑ HTC ↑ serum iron ↓ TIBC level ↑ EPO level -alleviated the hypertrophy of spleen -improved the liver coefficient	[65]
Aqueous extract of *Mangifera indica* stem bark	25, 50, and 75 mg/kg body weight	in vivo	iron deficiency anemia rat model	↑ PCV, Hb and RCB count ↑ lactase and sucrase activity	[66]
Aqueous extract of the stem bark of *Theobroma cacao* (TC)	25, 50, and 75 mg/kg body weight	in vivo	iron deficiency anemia model albino rats	↑ PCV, Hb and RCB count ↑ intestinal lactase and sucrase activity	[67]
*Caulis spatholobi* (CS)	cells treated with 400 mg/mL of extract AIN-76A diet containing 10.8% dried CS	in vitro in vivo	Human hepatocellular carcinoma cell lines Huh7 and HepG2 C57BL/6 mice diet containing 108 g dried CS per kilogram (10.8%)	↓ HAMP expression in Huh7 and HepG2 cells ↓ BMP-6-induced HAMP expression ↓ IL-6-induced HAMP expression ↑ iron mobilization in mice ↓ phosphorylation of Smad1/5/8 ↓ hepatic iron levels ↑ serum iron levels in mice	[68]
*Angelica sinensis* polysaccharide (ASP)	1 g/kg	in vivo	iron deficiency anemia in Sprague Dawley rats	↓ hepcidin expression	[50]
*Angelica sinensis* polysaccharide (ASP)	1 g kg^−1^	in vivo	iron deficiency anemia Sprague Dawley rats	↓ hepcidin expression	[51]
Beetroot juice (*Beta vulgaris* L.)	200 mL	clinical study	Twenty female soccer players 23.13 ± 0.77 years old	↑ Hb, HTC, RBC, iron, and ferritin levels ↓ TIBC	[58]
Beetroot juice (*Beta vulgaris* L.)	100 mL	clinical study	adolescent girls	↑ Hb	[59]
Beet powder (*Beta Vulgaris* L.) with Fe supplementation	8 g beetroot powder	clinical study	30 pregnant women with anemia	↑ Hb, HTC, ↑ the number of erythrocytes	[60]
*Carica papaya*	110 g of papaya	clinical study	42 pregnant women	↑ Hb ↑ HTC	[69]
Sweet potato (*Ipomoea Batatas* L.)	100 g	clinical study	first trimester pregnant women	↑ Hb	[70]
Baobab fruit *(Adansonia digitata)*	60, 80, and 100 g	clinical study	32 pregnant with iron deficiency anaemia	↑ Hb, PCV ↑ serum ferritin	[71]
Aqueous extract of *Hibiscus sabdariffa* seeds	400 mg of the extract/kg body weights		-nutritional anemia model -hemorrhagic anemia ratmodel	↑ HB, PCV, and RBC count of hemorrhagic anemic rats ↑ the Hb level of the nutritionally iron-deficient rats	[72]
Red beetroot (*Beta vulgaris* L.)	8 g	clinical study	healthy female volunteers (age range, 22 to 24)	↑ Hb ↓ TIBC ↑ ferritin ↓ transferrin ↑ serum iron levels	[57]

↑ increase/upregulated; ↓ decrease/downregulated; SUE, sulfate *Ulva polysaccharide*; RBCs, red blood cells; Hb, hemoglobin; TIBC, total iron-binding capacity; IL-4, interleukin 4; APS, *Astragalus membranaceus* polysaccharides; SOD, superoxide dismutase; CAT, catalase; MDA, malonyldialdehyde; TBARS, thiobarbituric acid reactive substances; LOOH, lipid hydroperoxide; GSH-Px, glutathione peroxidase; HTC, hematocrit; LPE, low-molecular-weight polysaccharide from *Enteromorpha prolifera*; EPO, erythropoietin; PCV, packed cell volume; TC, aqueous extract of the stem bark of *Theobroma cacao*; HAMP, hepcidin antimicrobial peptide; CS, *Caulis spatholobi*; ASP, *Angelica sinensis* polysaccharide; SMAD4, SMAD family member 4; BMP-6, bone morphogenic protein 6; FeSO_4_, ferrous sulfate.

**Table 2:** Pharmacological effects of the natural antioxidants in iron chelation activity.

Bioactive Compound	Doses	In Vitro/ In Vivo/ Clinical Study	Model	Bioactive Effect	References
EGCG and GSE		in vitro	cell culture of Caco-2 cells MEL cell culture mouse erythroleukemia (MEL) cell line	↓transepithelial heme iron transpor ↑ apical heme iron uptake (GSE) ↓ the cellular assimilation of heme (EGCG)	[83]
EGCG and GSE		in vitro	human Caco-2 (HTB-37TM) cell line murine erythroleukemia (MEL) cell line	↓heme iron absorption by reducing the basolateral iron export in Caco-2 cells	[108]
Curcumin	0.5% and 2.0%	in vivo	FVB mice treated with curcumin for 12 weeks	↓ in the H and L subunits of the iron storage protein ferritin ↑ transferrin receptor 1 ↑ activated iron-regulatory proteins (IRPs) ↓ in levels of ferritin in the liver	[88]
Curcumin	0.2%, 0.5%, and 2.0%	in vivo	C3H/HeNCrlmice AIN93M basal diet modified to contain various amounts of iron and curcumin: 5, 12, 50, or 1000 mg iron/kg diet plus curcumin at 0% (control), 0.2%, 0.5%, and 2.0% (wt/wt) for 26 weeks	↓ HTC, Hb, serum iron, and transferrin saturation ↓ iron levels in the bone marrow and spleen -appearance of hypochromic RBCs ↑ transferrin receptor 1 (TfR1) ↑ iron-responsive element-binding protein (IRP) ↓ ferritin ↓ hepcidin	[89]
Silybin		in vitro		-iron chelating role -high affinity for ferric ion -binds Fe(III) even at acidic pH	[105]
Silybin	140 mg silybin (Legalon Forte)	clinical study	patients with hereditary haemochromatosis	↓ postprandial iron absorption	[106]
Quercetin	100 mg/kg body weight	in vivo in vitro	ethanol-induced iron overload and liver damage in mice mouse primary hepatocytes	-attenuated the hepatic iron deposition in mice exposed to ethanol or excess iron -prevented ethanol induced hepatic iron overload by regulating hepcidin expression via the BMP6/SMAD4 signaling pathway	[109]
Myrecitin		in vitro	HepG2 an HEK293 cells	↓ HAMP mRNA levels ↓SMAD1/5/8 phosphorylation	[103]
Myrecitin	40-mg/kg myricetin daily for 1 or 5 days 10-mg/kg myricetin for 5 days, after which the mice were injected with LPS (5 mg/kg, ip) 0.2% (*w*/*w*) myricetin for up to 30 days	in vivo	C57BL/6 mice	↓ hepatic hepcidin expression ↓ splenic iron levels ↑ serum iron levels ↑ red blood cell counts ↑ Hb	[103]
Black soybean seed coat anthocyanins extract (BSSCE)	200 mg/mL BSSCE 2% BSSCE	in vitro in vivo	AIN-76A diet containing 2% BSSCE feeded to 8-week-old male C57BL/6 mice for 0, 1, 7, 15 or 30d	↓ hepcidin expression ↓ splenic Fe concentrations ↑ serum Fe concentrations ↑ in erythrocyte counts, Hb concentrations, HTC values	[110]
Citrus flavonoids-rich extracts from bergamot and orange juices		in vitro	iron overloaded human lung epithelial cells basal epithelial cell line A549, derived from human lung carcinoma	↓ ROS ↓ lipid peroxidation ↑mitochondrial function ↑ iron chelation -prevented DNA-oxidative damage in iron-exposed cells ↑ catalase activity	[111]
Baicalein (*Scutellaria baicalensis*)		in vitro		-induced iron chelation -inhibition of iron-mediated Fenton reaction via a combination of chelation and radical scavenging mechanism	[112]
Genistein		in vivo in vitro	-zebrafish embryo (*Danio* *rerio*) -the human hepatocarcinoma cell line, HepG2	↑ hepcidin expression and promoter activity in zebrafish and human hepatocytes in a STAT3- dependent and SMAD4-dependent manner	[113]
Vitamin C	50 and 100 mg/mL	in vivo	hepcidin-producing HepG2 cell line	-inhibition of hepcidin expression	[114]
Tucum-Do-Cerrado (*Bactris setosa* Mart.)	AIN-93G diet with 150 g of tucum-do-cerrado fruit (pulp and peel)/kg diet iron-supplemented rodent diet (350 mg of iron/kg diet) and 150 g of tucum-do-cerrado fruit/kg diet	in vivo	Wistar rats	↓ spleen lipid and protein oxidation; mRNA levels of hepatic Hamp and ferritin ↑ serum antioxidant capacity ↑ hepatic mRNA levels of BMP6, Hmox1, Nqo1, and Nrf2 -abrogated the liver Hamp iron-induced upregulation -prevented intestinal iron accumulation; hepatic lipid peroxidation; splenic protein damage ↑ of CAT, GR, and GSH-Px activity	[115]
*Angelica sinensis* polysaccharide (ASP)	ASP at 25, 50, and 100 mg kg^−1^	in vivo	BALB/c mice inoculated with H22 tumor cells	↓ hepcidin concentration in serum ↓ levels of serum IL-6 ↓ serum ferritin ↓ levels of serum Tf ↓ levels of serum TfR2 ↓ levels of serum TfR1 in the ASP 25 mg kg^−1^ treatment group	[116]
Hydro-alcoholic extract of *Medicago sativa* and *Allium porrum*	200 and 400 mg/kg body weight	in vivo	iron-overloaded rats	↓ serum ferritin ↓ serum iron level	[117]
Methanolic extract of Angel’s wings mushroom (*Pleurotus porrigens*)	200 and 400 mg/kg/24 h	in vivo	iron-overloaded mice NMRI mice	-chelation of excess iron ↓ in plasma Fe^3+^ content ↓ in the extent of necrotic hepatocytes, fibrous tissues, and pseudo-lobules	[118]
Methanol extract of *Nerium indicum* leaves	50, 100, and 200 mg/kg b.w.	in vivo	iron-overloaded mice	-antioxidant and iron-chelating properties ↓ iron overload induced toxicity -normalized the levels of ALAT, ASAT, ALP, and bilirubin ↑ levels of SOD, CAT, GST, and nonenzymatic-reduced glutathione ↓ levels of lipid peroxidation, protein carbonyl, hydroxyproline ↓ liver iron	[119]
*Emblica officinalis* fruit extract	50, 100, and 200 mg kg^−1^ body weight	in vivo	iron-overloaded Swiss albino mice	↓ liver iron ↓ serum ferritin ↓ ALAT, ASAT, ALP, and bilirubin ↓ in lipid peroxidation, protein oxidation, and collagen	[120]
Gallic acid (GA) and methyl gallate (MG) isolated from *Spondias pinnata* bark extract	2 and 4 mg/kg b.w. GA 2 and 4 mg/kg b.w. MG	in vitro in vivo	iron-overloaded Swiss albino mice	↓ ALAT, ASAT, ALP, and bilirubin ↓ ROS in liver and spleen homogenate ↓ ferritin-bound iron SOD, CAT, GST, GSH restioration	[121]
Aqueous and 70% methanol extracts of *Clerodendrum colebrookianum* leaves	50, 100 and 200 mg/kg body weight	in vitro in vivo	iron-overloaded Swiss albino mice	↓ liver iron ↓ serum ferritin ↓ ALAT, ASAT, ALP, and bilirubin SOD, CAT, GST, GSH restioration ↓ lipid peroxidation ↓ the protein carbonyl content	[122]
Methanol extract of *Terminalia chebula* Retz. fruit	50, 100, 200 mg/kg b.w.	in vitro in vivo	iron-overloaded Swiss albino mice	in vitro: iron chelation, and DNA protective effects in vivo: ↓ ALAT, ASAT, ALP, and bilirubin ↑ antioxidant acvtivity ↓ lipid peroxidation, protein carbonyl, hydroxyproline and liver iron ↓ iron overload-induced toxicity -potential activity for reductive release of ferritin iron	[123]
Baicalin and quercetin		in vitro in vivo	iron-dextran induced iron overload mice	in vitro study: released iron from ferritin In vivo: -inhibited iron overload induced lipid peroxidation and protein oxidation of liver ↓ hepatic iron and hepatic collagen content ↑ the serum non-heme iron level but not serum ferritin level ↑ the excretion of iron through feces	[124]
Mangiferin (M) and an aqueous leaf extract of *Mangifera foetida* L (EMF)	mangiferin 75 mg/kg EMF 2.930 g/kg	in vivo	iron-overloaded Sprague Dawley rats	↑ body weight ↓ plasma iron ↑ iron excretion via urine M and EMF antioxidant activity	[125]

EGCG (-), epigallocatechin-3-gallate; GSE, grape seed extract; MEL, mouse erythroleukemia; IRPs, iron-regulatory proteins; TfR1, transferrin receptor 1; IRP, iron-responsive element-binding protein; BSSCE, black soybean seed coat anthocyanins extract; VAD, vitamin A deficiency; IL-8, interleukin-8; ROS, reactive oxygen species; STAT3, signal transducer and activator of transcription 3; Tuc-AIN-93G added of tucum-do-cerrado; TucFe-AIN-93G with tucum-do-cerrado and iron-enriched; Hmox1, heme oxygenase 1; Nqo1, NADPH dehydrogenase quinone 1; Nrf2, factor nuclear factor-erythroid 2-related factor 2; GR, glutathione reductase; Tf, transferrin; TfR2, transferrin receptor 2; ALAT, alanine aminotransferase; ASAT, aspartate aminotransferase; ALP, alkaline phosphatase; GST, glutathione-S-transferase; GA, gallic acid; MG, methyl gallate; GSH, reduced glutathione; EMF, extract of *Mangifera foetida* L; HepG2, human hepatocellular carcinoma cells; HEK293, human embryonic kidney cells.

**Table 3:** Pharmacological effects of natural antioxidants in hemolytic anemia treatment.

Bioactive Compound	Doses	In Vitro/ In Vivo/ Clinical Study	Route of Administration	Model	Bioactive Effect	References
*Brassica oleraceae* var *italica* aqueous extract	100 and 200 mg/kg	in vivo	for 14 days	PHZ-induced anaemia in Sprague-Dawley rats	↑ RBC ↑ Hb	[128]
*Hypoestes triflora* aqueous extract	50 mg/kg	in vivo	orally for 30 days	phenylhydrazine hydrochloride-induced anaemia in guinea-pigs	↑ RBC count	[129]
*Phyllanthus niruri* Linn aqueous extract	250, 500, and 1000 mg/ kg	in vivo	orally using a feeding cannula once daily for 14 consecutive days	2,4-dinitrophenylhydrazine-induced haemolytic anaemia in Wister rats	↑ PCV, Hb, RBC concentration ↓ WBC, MCV, MHC and reticulocytes ↑ catalase and SOD activity	[130]
*Gossypium hirsutum* L. leaf ethanolic extract	100–400 mg/kg	in vivo	orally	PHZ-induced haemolytic anaemia in rats	↑ RBC, Hb, PCV, neutrophils and platelets ↓ WBC, lymphocytes, MCV, and MCH	[131]
*Falcaria vulgaris* leaf aqueous extract	25, 50, 100, and 200 mg/kg	in vivo	Orally For 15 days	PHZ-induced haemolytic anaemia in rats	↑ the levels of body weight, WBC, neutrophils, platelet, RBC, Hb, PCV, MCV, MCH, MCHC ↓ the raised levels of ALP, ASAT, ALAT, GGT, urea, creatinine, ferrous, ferritin, and erythropoietin	[132]
*Beta vulgaris* (beet) leaf aqueous extract	100 and 200 mg/kg	in vivo in vitro	Orally daily for 12 days	PHZ-induced haemolytic anemia model in albino rats	restored the levels of RBCs, WBCs, Hb, and HCT ↓ MCV ↑ MCHC ↓ MDA	[133]
*Pterocarpus erinaceus* stem bark aqueous extract	250 and 500 mg/kg	in vivo	Oral administration for 14 days	PHZ-induced anaemia in albino rats	↑ PCV, Hb, RBC, MCV, neutrophiles ↓ WBC, lymphocytes, platelets, MCH, MCHC ↓ ASAT, ALAT	[134]
*Lophira lanceolata* leaves aqueous extract	200, 400, and 800 mg/kg	in vivo	oral for 3 weeks	PHZ-induced anaemia in wistar rats	↑ in the RBC count, Hb concentration and PCV	[135]
*Mangifera indica* barck aqueous decoction	25, 50, and 100 mg/kg	in vivo	once daily for 14 consecutive days by oral feeding cannula	2,4-dinitrophenylhydrazine-induced haemolytic anaemia in Wistar rats	↑ PCV and Hb ↓ TLC	[136]
*Ficus sur* bark/fruit methanolic extract	50, 100, and 150 mg/Kg	in vivo	oral administration 14 days	PHZ-induced haemolytic anaemia in rats	↑ Hb, HTC, RBC count	[137]
*Justicea carnea* leaves ethanolic extract	500 and 1000 mg/kg	in vivo	orally gavage For 28 days	PHZ induced-anemia albino rats	↑ Hb, RBC, PCV ↓ cholesterol, triacylglycerol, and LDL cholesterol concentrations ↑ HDL-cholesterol	[138]
*Harungana madagascariensis* bark extract				PHZ and malaria parasites-induced anemia	↑ PCV, RBC and Hb	[139]
*Sorghum bicolor extract*	200 or 300 mg/kg	in vivo	gavage for 15 days	PHZ-induced anaemia in rats Wistar rats	↑ stimulation of Hb synthesis by activation of erythropoiesis ↑ MCV ↓ MCH -production and the early release of immature RBC	[140]
*Telfairia occidentalis leaf ethanolic extract*	200 mg/kg	in vivo	orally for 2 weeks	PHZ-induced anaemia in rats	↑ HCT, Hb, RBCs, lymphocytes, monocytes ↓ serum levels of total protein, albumin, globulin and bilirubin ↑ body weight	[141]
*Spinacia oleracea* leaf aqueous extract	100 mg/kg	in vivo	once daily for 28 days	PHZ-induced anaemia in rats	↑ Hb ↑ PCV ↑ RBC level	[142]
*Brillantasia nitens methanolic extract*	400, 800, 1600, 3200 mg/kg	in vivo	oral intubation daily for 4 weeks	PHZ-induced haemolytic anaemia in rats	↑ Hb, RBC, WBC and PVC	[143]
*Acacia nilotica ethanolic leaf extract*	100 mg/kg and 200 mg/kg	in vivo	orally for 14 days	PHZ-induced anaemia in rats	↑ Hb, RBC, WBC, HCT, PLT count	[144]
*Solanum nigrum* leaf methanolic extract	100, 200, 300, and 400 mg/kg	in vivo	orally by gastric intubation for three weeks	PHZ-induced anaemia in rats	↑ PVC, Hb, RBC, MCV, MCH, PLT ↓ WBC, lymphocytes and neutrophils -exhibited high radical scavenging activity	[145]
*Amaranthus cruentus ethanolic extract*	200 and 400 mg/kg	in vivo	for 15 days	PHZ-induced anemia in albino rats	↑ RBCs ↑ WBCs ↑ Hb ↑ HTC	[146]
*Mangifera indica* and *Telfairia occidentalis extracts*	20 mg kg	in vivo	oral daily dose	PHZ-induced anaemia in rabbits	↑ PCV values ↑ RBC counts ↑ Hb ↑ bilirubin	[147]
*Hibiscus sabdariffa* anthocyanins	100 mg/kg	in vivo	gavage for 4 weeks	2, 4-dinitrophenylhydrazine (2, 4-DNPH) rabbits	↑ in blood GSH ↑ RBC counts, PCV and Hb ↓ in MDA and WBC counts	[148]
*Justicia* *secunda* leaves extracts	200 mg/kg	in vivo	for 21 days	PHZ-induced anaemia in Sprague-Dawley rats	↑ the number of RBCs ↑Hb	[149]

PHZ, phenylhydrazine; WBC, white blood cell; MCV, mean corpuscular volume; MCH, mean corpuscular hemoglobin; MCHC, mean corpuscular hemoglobin concentration; GGT, gamma-glutamyl transferase; TLC, total leucocytes count; 2,4-DNPH, 2,4 dinitrophenylhydrazine.

**Table 4:** Pharmacological effects of natural antioxidants in β-thalassemia treatment.

Bioactive Compound	Doses	In Vitro/ In Vivo/ Clinical Study	Route of Administration	Model	Bioactive Effect	References
Resveratrol	5 μM	in vitro		CD34^+^ cells	↑ maturation of erythroid cells ↓ cell proliferation -induces cell differentiation of human β-thalassemic-erythroid cells	[169]
	2.5 mg/kg	in vivo	resveratrol incorporated into a standard diet for six months	β-thalassemia mice models	↓ ineffective erythropoiesis ↓ anemia ↑ HTC, Hb, MCV and MCH levels ↓ total bilirubin ↓ reticulocyte count ↓ oxidative damage in RBC ↑ RBC survival	[169]
Curcumin	500 mg daily for 12 months	clinical study		Beta-thalassemia/Hb E disease	↓ MDA, SOD, GSH-Px in RBC ↓ serum NTBI ↑ RBC GSH	[170]
Curcumin	100 μM curcumin	clinical study		Beta-thalassemia patients	↓ plasma NTBI	[171]
Fermented papaya preparation (FPP)	50 mg/kgdaily for 3 months	in vivo	Oral administration daily for 3 months	β-thalassemia mouse model	↑ GSH, PMN ↓ ROS ↓ lipid peroxidation ↓ externalization of phosphatidylserin	[172]
Green tea extract (GTE)	50 mg/kg EGCG	in vivo	for 2 months	β-knockout thalassemic (BKO) mice	↓ MDA, NTBI, and ALAT ↑ plasma hepcidin and insulin ↓ iron accumulation and MDA in pancreas and liver -improved liver and pancreatic β-cell activity by decreasing redox iron/free radicals	[173]
Curcumin	200 mg/kg and 50 mg/kg DFP	in vivo	for 2 months	mouse models	↓ plasma NTBI ↓ MDA concentrations ↓ heart iron accumulation -improved HRV	[174]
EGCG from green tea		in vitro		iron-treated erythrocytes	-bound Fe^3+^ and iron chelation ↓ oxidative stress	[175]
Extracts of green tea (GTE) and curcumin	17.3–35.5 mg/kgEGCG equivalent	clinical study	daily for 60 days	transfusion-dependent β-thalassemia (TDT) patients	↓ of blood urea nitrogen levels ↓ NTBI ↓ LPI -delayed in increasing lipid-peroxidation	[176]

FOxO3, Forkhead-box-class-O3; Prdx2, peroxiredoxin-2; NTBI, non-transferrin bound iron; FPP, fermented papaya preparation; PMN, polymorphonuclear; BKO, β-knockout thalassemic; CUR, curcuminoids; DFP, deferiprone; HRV, heart rate variability; LPI, labile plasma iron; ObEO, essential oil isolated from *Ocimum basilicum* L. leaves.

**Table 5:** Pharmacological effects of natural antioxidants in β-thalassemia major treatment.

Bioactive Compound	Doses	In Vitro/ In Vivo/ Clinical Study	Route of Administration	Model	Bioactive Effect	References
Silymarin	140 mg/kg + DFO	clinical study	three times/ day	59 patients with thalassemia major	↑ GSH levels of RBCs ↑ alkaline phosphatase ↓ serum iron and ferritin	[180]
Silymarin	Legalon capsules (140 mg)	clinical study	9 months	97 patients with β-thalassemia major	↓ ferritin and iron levels ↓ TIBC levels ↓ serum levels of hepcidin and soluble transferrin receptor (sTfR)	[166]
*Nigella sativa*	2 g/day	clinical study	*Nigella sativa* powder added to foods or drinks for 3 consecutive months	25 blood transfusion-dependent childrens with β-thalassemia major	↑ Hb, WBCs ↑ neutrophils ↓ MDA ↑ TAC	[181]
Fermented papaya	3 g	clinical study	3 g three times a day after meals for three months	patients with β-thal major	↑ GSH in RBCs ↓ ROS in RBCs ↓ lipid peroxidation	[182]
Curcumin	500 mg capsules (total: 1000 mg)	clinical study	twice daily for 12 weeks	68 β-thalassemia major patients	↓ NTBI ↓ ALAT, ASAT -alleviated iron burden and liver dysfunction	[183]
Green tea extract (GTE)	GTE+DFP (50 mg/kg)		daily orally for 3 months	β-thalassemic mice with iron overload	↓ plasma non-transferrin bound iron concentrations ↓ plasma ALAT activity ↓ tissue iron deposition ↓ plasma NTBI levels ↓ liver oxidative damage	[184]

DFO, deferoxamine; sTfR, soluble transferrin receptor; β-thal, β-thalassemia; PS, phosphatidylserine; GTE, green tea extract; DFP, deferiprone; TAC, total antioxidant activity.

**Table 6:** Pharmacological effects of natural antioxidants in sickle cell anemia treatment.

Bioactive Compound	Doses	In Vitro/ In Vivo/ Clinical Study	Route of Administration	Model	Bioactive Effect	References
DTT, N-NAC quercetin	0.25 mM DTT 10 mM N-NAC 100 µM quercetin	in vitro		sickle cell anemia in vitro model	-inhibition of the main cation pathways responsible for dehydration ↓ Ca^2+^-induced PS exposure and hemolysis ↓ RBCs fragility	[194]
Rutin		in silico in vitro		sickle erythrocytes induced with 2% metabisulphite	-restored the integrity of erythrocytes membrane -prevented and reversed lipid peroxidation ↑ GSH and CAT levels ↓ SOD activity	[199]
Chrysin		in silico in vitro		sickling was induced with 2% metabisulphite at 3 h.	-prevented sickling -re-established the integrity of erythrocytes membrane - prevented and reversed lipid peroxidation ↑ GSH, CAT ↓ SOD	[200]
*Pfaffia paniculata* extract	0.0, 0.2, or 0.5 mg/mL	in vitro		RBCs from patients with sickle cell disease	-improvement of RBC deformability in patients with SCD -the fragility of RBCs of patients with sickle cell disease was not affected	[201]
Aqueous extracts of *Dennettia tripetala* and *Physalis angulata* leaf extract		in vitro		homozygous sickle cell erythrocyte	↑ GSH, SOD ↓ catalase ↓ ROS ↓ % of sickled cells ↓ haemoglobin polymerization rate ↓ osmotic fragility of human sickle RBCs	[202]
Ethanol extract of *Terminalia catappa* L. (*Combretaceae*) leaves		in vitro		metabisulphite-induced sickling	-inhibited osmotically-induced hemolysis of human erythrocytes -prevented and reversed the sickling of human ‘SS’ erythrocythes	[203]
Extracts of the roots of *Cissus populnea* L.		in vitro		sodium metabisulphite induced sickling of the HbSS red blood cells	- anti-sickling activity	[204]
Methanolic leaf extracts of *Carica papaya* L. (*Caricaceae*)		in vitro		Hbss red blood cells obtained from non-crisis state sickle cell patients	↓ hemolysis and protected erythrocyte membrane integrity under osmotic stress conditions -inhibited formation of sickle cells under severe hypoxia	[205]
Leaves and stem of *Parquetina nigrescens* L.		in vitro		pre-sickled HbSS blood cell suspensions blood samples of noncrisis sickle cell individuals	-antisickling activity -protected the integrity of the erythrocyte membrane by the reduction in hemolysis of the Hbss cells -inhibited formation of sickle cells under severe hypoxia	[206]
Aqueous extract of *Carica papaya* leaf	2, 4, 6, 8, and 10 mg/mL				-prevented sickling - membrane stabilizing effect on HbSS red blood cells ↓ osmotic fragility of HbSS red blood cells	[207]
Divanilloylquinic acids isolated from *Fagara zanthoxyloides* Lam. (*Rutaceae*)				patients with severe sickle cell anemia	antisickling properties	[208]
Aqueous extract of *Zanthoxylum macrophylla*		in vitro		sodium metabisulphite-induced sickling in cells	-antisickling activity - stabilizing the RBC membranes	[209]
Aqueous extracts of *Zanthoxylum macrophylla* roots		in vitro		membrane preparations from human erythrocytes of HbAA, HbAS and HbSS bloods	modulation of the activities of the three membrane-bound ATPases: ↑ for Na^+^, K^+^- and Ca^2+^-ATPases ↓ for Mg^2+^-ATPase	[210]
Aqueous and methanolic extracts of leaves, seeds, and stem of *Telfairia occidentalis*	10 mg mL^−1^			sickled erythrocytes obtained from SCD patients	-aqueous leaves extract exhibited the highest antisickling activity - methanolic and aqueous stem extracts showed membrane stabilizing effects	[211]
Aqueous extracts of *Elaeis guineensis jacq* flowers					-anti-sickling activity ↓ the MCF values of the HbSS erythrocytes ↑ in Fe^2+^/Fe^3+^ ratio -altered the polymerization of sickle cell Hb -maintained erythrocyte membrane integrity	[212]
Methanol extract of *Mucuna pruriens* leaves	100, 200, 400, 600, and 800 mg/mL			sickle erythrocytes	- membrane stabilization ↓ haemolysis - scavenging activity of the DPPH and hydroxyl radical	[213]
Aqueous extracts of *Zanthoxyllum heitzii*	250, 500, and 1000 μg/mL	in vitro		metabisulfite (2%) induced- sickle erythrocytes	↓ % sickle cells -membrane cell stability -antioxidant activities	[214]
*Telferia occidentalis, Curcubit maxima, Curcumis sativum* and *Curcubit lonatus*		in vitro		sickle erythrocytes	-inhibited sickle cell Hb polymerization -improved Fe^2+^/Fe^3+^ ratio	[215]
*Solenostemon monostachyus* (SolMon), *Carica papaya* seed oil (Cari-oil) and *Ipomoea involucrata* (Ipocrata)		in vitro		sickle erythrocytes	↓ % sickle cells ↑ RBCs ↓ Fe^2+^/Fe^3+^ ratio -inhibited sickle cell polymerization ↓ LDH ↑ CAT activity	[216]
Ethanol extract of *Annona Muricata, Delonix Regia* and *Senna Alata*		in vitro		sickle erythrocytes	↑ GHS and CAT activities ↓ SOD activity ↑ in membrane stability ↓ the sickling activity ↓ the polymerization of sickle cells	[217]
Ursolic acid isolated from the leaves of *Ocimum gratissimum* L.		in vitro		sickle erythrocytes	- anti-sickling effects ↑ RBCs	[218]
Extracts of the seed, flower and leaf of *Moringa oleifera*		in vitro		sickle erythrocytes	- anti-sickling effects	[219]
Aqueous methanol extracts of *Scoparia dulcis* and fractions	100, 300 and 500 mg/m extract 500 mg/mL fractions	in vitro in vivo	daily administration of the extract for 30 days	sickle erythrocytes Swiss albino mice and Wistar rats	- anti-sickling effects	[220]
Aqueous extract of unripe pawpaw (*Carica papaya*)				sickle cells of patients	- prevented sickling of Hb SS red cells and reversed sickled Hb SS red cells	[221]
Leaves ethanol extracts of *Hymenocardia acida* Tul (Euphorbiaceae)				sickle erythrocytes	-reversed sickled human RBC -the fractions containing flavonoids, saponins and carboxylic acids were found to be responsible for reversal of the sickled RBC.	[222]
Leaf and gel extracts of the Aloe vera (*Aloe barbadensis*)		in vitro		sickle erythrocytes	-inhibited sickle cell polymerization -improved of the Fe^2+^/Fe^3+^ ratio of HbSS	[223]
Aged garlic extract	5 mL daily	clinical study	for 4 weeks	five patients with sickle-cell anemia	↓ number of Heinz bodies -antioxidant activity on sickle RBCs	[224]
of *Moringa Oleifera* Seeds and leaves extracts		in vitro		erythrocyte cells deoxygenated with 2% sodium metabisulphite	-antisickling activity in deoxygenated erythrocytes	[225]
*Amphimas pterocarpoides*		in vitro		the sickling of RBCs was induced using sodium metabisulfite (2%)	- anti-sickling effects ↑ the solubility of the deoxy-haemoglobin S -allowed the rehydration of SS cells by reinforcing their capacity to resist against osmotic fragility	[226]
Isoquercitrin		in vitro		sickle erythrocytes	↓ % sickle cells ↓poimerization ↑ the oxygen affinity ↑ osmotic fragility of the sickle RBCs	[227]
*Hyphaene Thebaica* (Doum) fruit extract	1000, 500, and 250 μg/mL	in vitro		incubation of RBCs with 2% sodium metabisulte	↑ in the percentage of unsickled RBCs	[228]
Genistein		in vitro		sickle erythrocytes	↓ polymerization of Hb S ↓ % sickle cells ↑ the osmotic fragility of the erythrocyte cell	[229]
Methanol seed extract of *Buchholzia coriacea* and *Mucuna pruriens* seed extract	50%, 25%, 12.5%, and 6.25% seed extracts	in vitro		sickle cell blood from sickle cell disease patients with subsequent addition of 2% sodium metabisulphite to cause more sickling.	*Buchholzia coriacea:* -inhibited sickling -reversed sickled RBC -inhibited polymerisation *Mucuna pruriens*: ↑ the solubility of sickle Hb ↑ Fe^2+^/Fe^3+^ ratio ↓ osmotic fragility	[230]

DTT, dithiothreitol; NAC, N-acetylcysteine; 2,3-BPG, 2,3-bisphosphoglycerate; SCD, sickle cell disease; MCF, mean corpuscular fragility; DPPH-2, 2-diphenyl-l-picryl hydrazyl; SolMon, *Solenostemon monostachyus*; Cari-oil, *Carica papaya* seed oil; Ipocrata, *Ipomoea involucrata*; EFCM, Mbalmayo/Ebolowa cocoa bean extract; EFCB, Bertoua cocoa bean extract; FRAP, ferric-reducing antioxidant power.

**Table 7:** Pharmacological effects of natural antioxidants in aplastic anemia treatment.

Bioactive Compound	Doses	In Vitro/ In Vivo/ Clinical Study	Route of Administration	Model	Bioactive Effect	References
Leaf *Panax notoginseng* saponins (LPNS)	50, 100, and 200 mg/kg	In vivo	intragastric administration daily for 14 days	Aplastic anemia model in BALB/c mice	↑ WBC, platelets, RBC, Hb ↑ hematopoiesis -improve myelosuppression ↓ inflammation	[247]
Curcumin and baicalein	-curcumin (1 and 4 g/kg) -baicalein (0.6 and 2 g/kg) dissolved in corn oil	In vivo	intragastric administration once a day for 5 weeks	Aplastic anemia mouse model with iron overload	↑ WBC ↑ Hb levels ↑ hepcidin and its regulators (BMP-6, SMAD4, and TfR2)	[248]
Ginsenoside Rb1	1, 2, and 4 mg/mL	In vivo	intraperitoneally injection daily for 12 days	An immune-mediated aplastic anemia mouse model	↑ WBC, HGB, PLT, and bone marrow stem cells ↓ T-cell activation by suppressing DC maturation	[249]
Panaxadiol saponins component	20, 40, and 80 mg/kg	In vivo	intragastric daily for 15 days	Aplastic anemia model mice BALB/c mice	↑ WBC, platelet, neutrophil counts -enhanced proliferation of hematopoietic progenitor cells ↑ peripheral blood CD3^+^ and CD3^+^CD4^+^ cells and ↓ CD3^+^CD8^+^ cells ↑ CD4^+^CD25^+^FoxP3^+^ cells,	[250]
Saponins from *Dioscorea nipponica*	37.44, 74.88, and 149.76 mg/kg	In vivo	orally for 14 days	Aplastic anemia model mice	-alleviated pancytopenia with a hypocellular bone marrow ↑ the percentage of CD4^+^ cells in BMNC ↑ the CD4^+^/CD8^+^ ratio ↓ the pro-inflammatory cytokine concentrations of IL-2 and IFN-γ ↑the anti-inflammatory cytokine IL-4 -inhibited Fas–FasL-induced BMNC apoptosis -suppressed intracellular apoptosis	[251]

LPNS, leaf *Panax notoginseng* saponins; BMP-6, bone morphogenic protein 6; HGB, hematocrit and hemoglobin; HPC, hematopoietic progenitor cells; CFU-GM, colony-forming unit for granulocytes and macrophages; CFU-E, colony forming unit-erythroid; BMNC, bone marrow nucleated cells; IFN-γ, interferon-γ; GATA-3, erythroid transcription factor-3.

## References

[1] Kumar S., Anukiruthika T., Dutta S., Kashyap A.V., Moses J.A., Anandharamakrishnan C. (2020). Iron deficiency anemia: A comprehensive review on iron absorption, bioavailability and emerging food fortification approaches. Trends Food Sci. Technol..

[2] World Health Organization (2011). Haemoglobin Concentrations for the Diagnosis of Anaemia and Assessment of Severity. https://www.who.int/vmnis/indicators/haemoglobin.pdf.

[3] Rhodes C.E., Varacallo M. (2020). Physiology, oxygen transport. StatPearls.

[4] Balarajan Y., Ramakrishnan U., Özaltin E., Shankar A.H., Subramanian S.V. (2011). Anaemia in low-income and middle-income countries. Lancet.

[5] McLean E., Cogswell M., Egli I., Wojdyla D., de Benoist B. (2009). Worldwide prevalence of anaemia, WHO vitamin and mineral nutrition information system, 1993–2005. Publ. Health Nutr..

[6] Figueiredo A.C.M.G., Gomes-Filho I.S., Silva R.B., Pereira P.P.S., Mata F.A.F.D., Lyrio A.O., Souza E.S., Cruz S.S., Pereira M.G. (2018). Maternal anemia and low birth weight: A systematic review and meta-analysis. Nutrients.

[7] Haider B.A., Olofin I., Wang M., Spiegelman D., Ezzati M., Fawzi W.W. (2013). Anaemia, prenatal iron use, and risk of adverse pregnancy outcomes: Systematic review and meta-analysis. BMJ.

[8] Rahman M.M., Abe S.K., Rahman M.S., Kanda M., Narita S., Bilano V., Ota E., Gilmour S., Shibuya K. (2016). Maternal anemia and risk of adverse birth and health outcomes in low- and middle-income countries: Systematic review and meta-analysis. Am. J. Clin. Nutr..

[9] McCann J.C., Ames B.N. (2007). An overview of evidence for a causal relation between iron deficiency during development and deficits in cognitive or behavioral function. Am. J. Clin. Nutr..

[10] Hillman R.S., Adult K.A., Leporrier M., Rinder H.M., Hillman R.S., Ault K.A. (2001). Clinical approach to anemia. Hematology in Clinical Practice.

[11] GBD 2015 Disease and Injury Incidence and Prevalence Collaborators (2016). Global, regional, and national incidence, prevalence, and years lived with disability for 310 diseases and injuries, 1990–2015: A systematic analysis for the Global Burden of Disease Study 2015. Lancet.

[12] Moreno Chulilla J.A., Romero Colás M.S., Gutiérrez Martín M. (2009). Classification of anemia for gastroenterologists. World J. Gastroenterol..

[13] Rozman C., Feliu E., Grañena A., Monserrat E., Vives Corrons J.L. (1981). Hematologia. Atlas Practico Para El Medico General.

[14] Zucker S., Friedman S., Lysik R.M. (1974). Bone marrow erythropoiesis in the anemia of infection, inflammation, and malignancy. J. Clin. Invest..

[15] Chaudhry H.S., Kasarla M.R. (2020). Microcytic hypochromic anemia. StatPearls.

[16] Fenta D.A., Nuru M.M., Yemane T., Asres Y., Wube T.B. (2020). Anemia and related factors among highly active antiretroviral therapy experienced children in Hawassa Comprehensive Specialized Hospital, Southern Ethiopia: Emphasis on Patient Management. Drug Healthc. Patient Saf..

[17] Lanier J.B., Park J.J., Callahan R.C. (2018). Anemia in older adults. Am. Fam. Physician.

[18] Válka J., Čermák J. (2018). Differential diagnosis of anemia. Vnitri Lek..

[19] Sen Gupta A. (2019). Hemoglobin-based oxygen carriers: Current state-of-the-art and novel molecules. Shock.

[20] Patel S., Jose A., Mohiuddin S.S. (2020). Physiology, oxygen transport and carbon dioxide dissociation curve. StatPearls.

[21] Gafter-Gvili A., Schechter A., Rozen-Zvi B. (2019). Iron deficiency anemia in chronic kidney disease. Acta Haematol..

[22] Gómez-Ramírez S., Bisbe E., Shander A., Spahn D.R., Muñoz M. (2019). Management of perioperative iron deficiency anemia. Acta Haematol..

[23] Rodgers G.M., Gilreath J.A. (2019). The role of intravenous iron in the treatment of anemia associated with cancer and chemotherapy. Acta Haematol..

[24] Chuncharunee S., Teawtrakul N., Siritanaratkul N., Chueamuangphan N. (2019). Review of disease-related complications and management in adult patients with thalassemia: A multi-center study in Thailand. PLoS ONE.

[25] DeLoughery T.G. (2019). Safety of oral and intravenous iron. Acta Haematol..

[26] Ems T., St Lucia K., Huecker M.R. (2020). Biochemistry, iron absorption. StatPearls.

[27] Camaschella C. (2015). Iron-deficiency anemia. N. Engl. J. Med..

[28] Coyne D.W., Auerbach M. (2010). Anemia management in chronic kidney disease: Intravenous iron steps forward. Am. J. Hematol..

[29] Qi X., Zhang Y., Guo H., Hai Y., Luo Y., Yue T. (2020). Mechanism and intervention measures of iron side effects on the intestine. Crit. Rev. Food Sci. Nutr..

[30] Knutson M.D., Walter P.B., Ames B.N., Viteri F.E. (2000). Both iron deficiency and daily iron supplements increase lipid peroxidation in rats. J. Nutr..

[31] Connor J., Beard J.L. (1997). Dietary iron supplements in the elderly: To use or not to use?. Nutr. Today.

[32] Beard J.L., Dawson H., Pinero D. (1996). Iron metabolism: A comprehensive review. Nutr. Rev..

[33] Beard J.L. (1993). Are we at risk for heart disease because of normal iron status?. Nutr. Rev..

[34] Srigiridhar K., Nair K.M., Subramanian R., Singotamu L. (2001). Oral repletion of iron induces free radical mediated alterations in the gastrointestinal tract of rat. Mol. Cell Biochem..

[35] Schulz J.B., Lindenau J., Seyfried J., Dichgans J. (2000). Glutathione, oxidative stress and neurodegeneration. Eur. J. Biochem..

[36] Kontoghiorghe C.N., Kolnagou A., Kontoghiorghes G.J. (2015). Phytochelators intended for clinical use in iron overload, other diseases of iron imbalance and free radical pathology. Molecules.

[37] Varoni E.M., Lodi G., Iriti M. (2015). Efficacy behind activity-phytotherapeutics are not different from pharmaceuticals. Pharm. Biol..

[38] Trivedi S., Pandey R. (2020). 5′-Hydroxy-6, 7, 8, 3′, 4′-pentamethoxyflavone extends longevity mediated by DR-induced autophagy and oxidative stress resistance in C. elegans. Geroscience.

[39] Macciò A., Madeddu C. (2012). Management of anemia of inflammation in the elderly. Anemia.

[40] Warner M.J., Kamran M.T. (2020). Iron deficiency anemia. StatPearls.

[41] Lei M., Xue C.H., Wang Y.M., Li Z.J., Xue Y., Wang J.F. (2008). Effect of squid ink melanin-Fe on iron deficiency anemia remission. J. Food Sci..

[42] Zhang Z.S., Wang X.M., Han Z.P., Yin L., Zhao M.X., Yu S.C. (2012). Physicochemical properties and inhibition effect on iron deficiency anemia of a novel polysaccharide-iron complex (LPPC). Bioorg. Med. Chem. Lett..

[43] Hudson J.Q., Comstock T.J. (2001). Considerations for optimal iron use for anemia due to chronic kidney disease. Clin. Ther..

[44] Somsook E., Hinsin D., Buakhrong P., Teanchai R., Mophan N., Pohmakotr M., Shiowatana J. (2005). Interactions between iron(III) and sucrose, dextran, or starch in complexes. Carbohydr. Polym..

[45] Li Y., Cui J., Zhang G., Liu Z., Guan H., Hwang H., Aker W.G., Wang P. (2016). Optimization study on the hydrogen peroxide pretreatment and production of bioethanol from seaweed *Ulva prolifera* biomass. Bioresour. Technol..

[46] Qi X., Mao W., Gao Y., Chen Y., Chen Y., Zhao C., Li N., Wang C., Yan M., Lin C. (2012). Chemical characteristic of an anticoagulant-active sulfated polysaccharide from *Enteromorpha clathrata*. Carbohydr. Polym..

[47] Li Y., Wang X., Jiang Y., Wang J., Hwang H., Yang X., Wang P. (2019). Structure characterization of low molecular weight sulfate *Ulva polysaccharide* and the effect of its derivative on iron deficiency anemia. Int. J. Biol. Macromol..

[48] Sarker S.D., Nahar L. (2004). Natural medicine: The genus Angelica. Curr. Med. Chem..

[49] Wang P.P., Zhang Y., Dai L.Q., Wang K.P. (2007). Effect of *Angelica sinensis* polysaccharide-iron complex on iron deficiency anemia in rats. Chin. J. Integr. Med..

[50] Liu J.Y., Zhang Y., You R.X., Zeng F., Guo D., Wang K.P. (2012). Polysaccharide isolated from *Angelica sinensis* inhibits hepcidin expression in rats with iron deficiency anemia. J. Med. Food.

[51] Zhang Y., Cheng Y., Wang N., Zhang Q., Wang K. (2014). The action of JAK, SMAD and ERK signal pathways on hepcidin suppression by polysaccharides from *Angelica sinensis* in rats with iron deficiency anemia. Food Funct..

[52] Raman A., Lin Z.X., Sviderskaya E., Kowalska D. (1996). Investigation of the effect of *Angelica sinensis* root extract on the proliferation of melanocytes in culture. J. Ethnopharmacol..

[53] Wang Y., Zhu B. (1996). The effect of *Angelica polysaccharide* on proliferation and differentiation of hematopoietic progenitor cell. Zhonghua Yi Xue Za Zhi.

[54] Kale R., Sawate A., Kshirsagar R., Patil B., Mane R. (2018). Studies on evaluation of physical and chemical composition of beetroot (*Beta vulgaris* L.). Int. J. Chem. Stud..

[55] Clifford T., Howatson G., West D.J., Stevenson E.J. (2015). The potential benefits of red beetroot supplementation in health and disease. Nutrients.

[56] Priya N.G., Malarvizhi M., Jothi A.J. (2013). Beet root juice on haemoglobin among adolescent girls. IOSR J. Nurs. Health Sci..

[57] Al-aboud N.M. (2018). Effect of red beetroot (*Beta vulgaris* L.) intake on the level of some hematological tests in a group of female volunteers. ISABB. J. Food Agric. Sci..

[58] Lotfi M., Azizi M., Tahmasbi W., Bashiri P. (2018). The effects of consuming 6 weeks of beetroot juice (*Beta vulgaris* L.) on hematological parameters in female soccer players. J. Kermanshah Univ. Med. Sci..

[59] Kavitha S.R., Dinesh K. (2020). An experimental study to determine the effectiveness of beetroot juice on hemoglobin among girls of selected hostel girls, Bidar, Karnataka. World J. Adv. Healthc. Res..

[60] Triana H., Hadisaputro S., Djamil M. (2020). Effect of beet powder (*Beta Vulgaris* L.) with Fe supplementation on increasing hemoglobin, hematocrit, and erythrocyte levels in pregnant women with anemia. STRADA J. Ilm. Kesehat..

[61] Saini R.K., Manoj P., Shetty N.P., Srinivasan K., Giridhar P. (2014). Dietary iron supplements and *Moringa oleifera* leaves influence the liver hepcidin messenger RNA expression and biochemical indices of iron status in rats. Nutr. Res..

[62] Jia N., Qiao H., Zhu W., Zhu M., Meng Q., Lu Q., Zu Y. (2019). Antioxidant, immunomodulatory, oxidative stress inhibitory and iron supplementation effect of *Astragalus membranaceus* polysaccharide-iron (III) complex on iron-deficiency anemia mouse model. Int. J. Biol. Macromol..

[63] Kulkarni R., Deshpande A., Saxena K., Varma M., Sinha A.R., Aurobindo S. (2012). Ginger supplementary therapy for iron absorption in iron deficiency anemia. Indian J. Tradit. Knowl..

[64] Mazhar M., Kabir N., Simjee S.U. (2018). Quercetin modulates iron homeostasis and iNOS expression of splenic macrophages in a rat model of iron deficiency anemia. Chin. J. Nat. Med..

[65] Cui J., Li Y., Yu P., Zhan Q., Wang J., Chi Y., Wang P. (2018). A novel low molecular weight *Enteromorpha polysaccharide*-iron (III) complex and its effect on rats with iron deficiency anemia (IDA). Int. J. Biol. Macromol..

[66] Modupe O., Oladiji T.A. (2016). Optimizing dose of aqueous extract of *Mangifera indica* L stem bark for treating anaemia and its effect on some disaccharidases activity in iron deficient weanling rats. J. Nutr. Intermed. Metab..

[67] Modupe O., Olupo A.O., Oladiji T.A. (2018). Dose-dependent effects of *Theobroma cacao* in iron deficient anemia treatment in rats. J. Nutr. Intermed. Metab..

[68] Guan Y., An P., Zhang Z., Zhang F., Yu Y., Wu Q., Wang F. (2013). Screening identifies the Chinese medicinal plant *Caulis spatholobi* as an effective HAMP expression inhibitor. J. Nutr..

[69] Eliagita C., Kuntjoro T., Sumarni S., Suwondo A., Hadisaputro S., Eliagita C., Mulyantoro D.K. (2017). Effect of consuming papaya (*Carica papaya* Linn.) on the level of hemoglobin and hematocrit in pregnant women with anemia. Belitung Nurs. J..

[70] Felina M. (2019). Sweet potatoes consumption on increased hemoglobin levels in first trimester pregnant women. Bloss. J. Midwifery.

[71] Kheiri N.A., Sharfi I.Y. (2006). The anti-anaemic properties of Baobab fruit (*Adansonia digitata*). FRC J. Food. Sci. Technol..

[72] Ahmed R.H., Mohammed A.W.H., Hadi H.M.E., Khalifa M.Y.E. (2013). The effect of aqueous extract of *Hibiscus sabdariffa* seeds on hematological parameters of anemic rats. Int. J. Phytomed..

[73] Cappellini M.D. (2007). Exjade(R) (deferasirox, ICL670) in the treatment of chronic iron overload associated with blood transfusion. Ther. Clin. Risk Manag..

[74] Ozment C.P., Turi J.L. (2009). Iron overload following red blood cell transfusion and its impact on disease severity. Biochim. Biophys. Acta.

[75] Piomelli S. (1995). The management of patients with Cooley’s anemia: Transfusions and splenectomy. Semin. Hematol..

[76] Hezaveh Z.S., Shidfar F. (2019). Hydrophilic phytochelators in iron overload condition. J. Nutr. Food Secur..

[77] Vanhees K., Godschalk R.W., Sanders A., van Doorn S.B.V.W., van Schooten F.J. (2011). Maternal quercetin intake during pregnancy results in an adapted iron homeostasis at adulthood. Toxicology.

[78] Petry N., Watson R.R., Preedy V.R., Zibadi S. (2014). Polyphenols and low iron bioavailability. Polyphenols in Human Health and Disease.

[79] Lesjak M., Hoque R., Balesaria S., Skinner V., Debnam E.S., Srai S.K., Sharp P.A. (2014). Quercetin inhibits intestinal iron absorption and ferroportin transporter expression in vivo and in vitro. PLoS ONE.

[80] Mladěnka P., Macáková K., Filipský T., Zatloukalová L., Jahodář L., Bovicelli P., Silvestri I.P., Hrdina R., Saso L. (2011). In vitro analysis of iron chelating activity of flavonoids. J. Inorg. Biochem..

[81] Kim E.Y., Ham S., Bradke D., Ma Q., Han O. (2011). Ascorbic acid offsets the inhibitory effect of bioactive dietary polyphenolic compounds on transepithelial iron transport in Caco-2 intestinal cells. J. Nutr..

[82] Lesjak M., Balesaria S., Skinner V., Debnam E.S., Srai S.K.S. (2019). Quercetin inhibits intestinal non-haem iron absorption by regulating iron metabolism genes in the tissues. Eur. J. Nutr..

[83] Ma Q., Kim E.Y., Han O. (2010). Bioactive dietary polyphenols decrease heme iron absorption by decreasing basolateral iron release in human intestinal Caco-2 cells. J. Nutr..

[84] Guo Q., Zhao B., Li M., Shen S., Xin W. (1996). Studies on protective mechanisms of four components of green tea polyphenols against lipid peroxidation in synaptosomes. Biochim. Biophys. Acta.

[85] Apak R., Guclu K., Ozyurek M., Karademir S.E. (2004). Novel total antioxidant capacity index for dietary polyphenols and vitamins C and E, using their cupric ion reducing capability in the presence of neocuproine: CUPRAC method. J. Agric. Food Chem..

[86] Kim E.Y., Ham S.K., Shigenaga M.K., Han O. (2008). Bioactive dietary polyphenolic compounds reduce nonheme iron transport across human intestinal cell monolayers. J. Nutr..

[87] Sharifi-Rad J., Rayess Y.E., Rizk A.A., Sadaka C., Zgheib R., Zam W., Sestito S., Rapposelli S., Neffe-Skocińska K., Zielińska D. (2020). Turmeric and Its Major Compound Curcumin on Health: Bioactive Effects and Safety Profiles for Food, Pharmaceutical, Biotechnological and Medicinal Applications. Front. Pharmacol..

[88] Jiao Y., Wilkinson J., Christine Pietsch E., Buss J.L., Wang W., Planalp R., Torti F.M., Torti S.V. (2006). Iron chelation in the biological activity of curcumin. Free Radic. Biol. Med..

[89] Jiao Y., Wilkinson J., Di X., Wang W., Hatcher H., Kock N.D., Torti S.V. (2009). Curcumin, a cancer chemopreventive and chemotherapeutic agent, is a biologically active iron chelator. Blood.

[90] Day A.J., Cañada F.J., Díaz J.C., Kroon P.A., Mclauchlan R., Faulds C.B., Plumb G.W., Morgan M.R., Williamson G. (2000). Dietary flavonoid and isoflavone glycosides are hydrolysed by the lactase site of lactase phlorizin hydrolase. FEBS Lett..

[91] Kroon P.A., Clifford M.N., Crozier A., Day A.J., Donovan J.L., Manach C., Williamson G. (2004). How should we assess the effects of exposure to dietary polyphenols in vitro?. Am. J. Clin. Nutr..

[92] Scalbert A., Morand C., Manach C., Rémésy C. (2002). Absorption and metabolism of polyphenols in the gut and impact on health. Biomed. Pharmacother..

[93] Hämäläinen M., Nieminen R., Vuorela P., Heinonen M., Moilanen E. (2007). Anti-inflammatory effects of flavonoids: Genistein, kaempferol, quercetin, and daidzein inhibit STAT-1 and NF-kappaB activations, whereas flavone, isorhamnetin, naringenin, and pelargonidin inhibit only NF-kappaB activation along with their inhibitory effect on iNOS expression and NO production in activated macrophages. Mediat. Inflamm..

[94] Bayele H.K., Balesaria S., Srai S.K.S. (2015). Phytoestrogens modulate hepcidin expression by Nrf2: Implications for dietary control of iron absorption. Free Radic. Biol. Med..

[95] Granado-Serrano A.B., Martín M.A., Bravo L., Goya L., Ramos S. (2012). Quercetin modulates Nrf2 and glutathione-related defenses in HepG2 cells: Involvement of p38. Chem. Biol. Interact..

[96] Lesjak M., Srai S.K.S. (2019). Role of Dietary Flavonoids in Iron Homeostasis. Pharmaceuticals.

[97] Leopoldini M., Russo N., Toscano M. (2011). The molecular basis of working mechanism of natural polyphenolic antioxidants. Food Chem..

[98] Comporti M., Signorini C., Buonocore G., Ciccoli L. (2002). Iron release, oxidative stress and erythrocyte ageing. Free Radic. Biol. Med..

[99] Ferrali M., Signorini C., Caciotti B., Sugherini L., Ciccoli L., Giachetti D., Comporti M. (1997). Protection against oxidative damage of erythrocyte membrane by the flavonoid quercetin and its relation to iron chelating activity. FEBS Lett..

[100] Vlachodimitropoulou E., Sharp P.A., Naftalin R.J. (2011). Quercetin-iron chelates are transported via glucose transporters. Free Radic. Biol. Med..

[101] Perron N.R., Brumaghim J.L. (2009). A Review of the Antioxidant Mechanisms of Polyphenol Compounds Related to Iron Binding. Cell Biochem. Biophys..

[102] Mira L., Fernandez M.T., Santos M., Rocha R., Florencio M.H., Jennings K.R. (2002). Interactions of flavonoids with iron and copper ions: A mechanism for their antioxidant activity. Free Radic. Res..

[103] Mu M., An P., Wu Q., Shen X., Shao D., Wang H., Wang F. (2016). The dietary flavonoid myricetin regulates iron homeostasis by suppressing hepcidin expression. J. Nutr. Biochem..

[104] Gharagozloo M., Khoshdel Z., Amirghofran Z. (2008). The effect of an iron (III) chelator, silybin, on the proliferation and cell cycle of Jurkat cells: A comparison with desferrioxamine. Eur. J. Pharmacol..

[105] Borsari M., Gabbi C., Ghelfi F., Grandi R., Saladini M., Severi S., Borella F. (2001). Silybin, a new iron-chelating agent. J. Inorg. Biochem..

[106] Hutchinson C., Bomford A., Geissler C.A. (2010). The iron-chelating potential of silybin in patients with hereditary haemochromatosis. Eur. J. Clin. Nutr..

[107] Bares J.M., Berger J., Nelson J.E., Messner D.J., Schildt S., Standish L.J., Kowdley K.V. (2008). Silybin treatment is associated with reduction in serum ferritin in patients with chronic hepatitis C. J. Clin. Gastroenterol..

[108] Ma Q., Kim E.Y., Lindsay E.A., Han O. (2011). Bioactive dietary polyphenols inhibit heme iron absorption in a dose-dependent manner in human intestinal Caco-2 Cells. J. Food Sci..

[109] Tang Y., Li Y., Yu H., Gao C., Liu L., Chen S., Yao P. (2014). Quercetin prevents ethanol-induced iron overload by regulating hepcidin through the BMP6/SMAD4 signaling pathway. J. Nutr. Biochem..

[110] Mu M., Wu A., An P., Du X., Wu Q., Shen X., Wang F. (2014). Black soyabean seed coat extract regulates iron metabolism by inhibiting the expression of hepcidin. Br. J. Nutr..

[111] Ferlazzo N., Visalli G., Cirmi S., Lombardo G.E., Laganà P., Di Pietro A., Navarra M. (2016). Natural iron chelators: Protective role in A549 cells of flavonoids-rich extracts of Citrus juices in Fe^3+^-induced oxidative stress. Environ. Toxicol. Pharmacol..

[112] Perez C.A., Wei Y., Guo M. (2009). Iron-binding and anti-Fenton properties of baicalein and baicalin. J. Inorg. Biochem..

[113] Zhen A.W., Nguyen N.H., Gibert Y., Motola S., Buckett P., Wessling-Resnick M., Fraenkel P.G. (2013). The small molecule, genistein, increases hepcidin expression in human hepatocytes. Hepatology.

[114] Chiu P.F., Ko S.Y., Chang C.C. (2012). Vitamin C affects the expression of hepcidin and erythropoietin receptor in HepG2 cells. J. Ren. Nutr..

[115] Fustinoni-Reis A.M., Arruda S.F., Dourado L.P., da Cunha M.S., Siqueira E. (2016). Tucum-Do-Cerrado (Bactrissetosa Mart.) consumption modulates iron homeostasis and prevents iron-induced oxidative stress in the rat liver. Nutrients.

[116] Cheng Y., Zhou J., Li Q., Liu Y., Wang K., Zhang Y. (2016). The effects of polysaccharides from the root of *Angelica sinensis* on tumor growth and iron metabolism in H22-bearing mice. Food Funct..

[117] Mirzaei A., Delaviz H., Mirzaei M., Toloei M. (2015). The effects of *Medicago sativa* and *Allium porrum* on iron overload in rats. Glob. J. Health Sci..

[118] Khalili M., Ebrahimzadeh M.A., Kosaryan M. (2015). In vivo iron-chelating activity and phenolic profiles of the angel’s wings mushroom, *Pleurotus porrigens* (Higher Basidiomycetes). Int. J. Med. Mushrooms.

[119] Ghate N.B., Chaudhuri D., Panja S., Mandal N. (2015). *Nerium indicum* leaf alleviates iron-induced oxidative stress and hepatic injury in mice. Pharm. Biol..

[120] Sarkar R., Hazra B., Mandal N. (2015). Amelioration of iron overload-induced liver toxicity by a potent antioxidant and iron chelator, *Emblica officinalis* Gaertn. Toxicol. Ind. Health.

[121] Chaudhuri D., Ghate N.B., Panja S., Mandal N. (2016). Role of phenolics from *Spondias pinnata* bark in amelioration of iron overload induced hepatic damage in Swiss albino mice. BMC Pharmacol. Toxicol..

[122] Das A., Chaudhuri D., Ghate N.B., Panja S., Chatterjee A., Mandal N. (2015). Protective effect of *Clerodendrum colebrookianum* leaves against iron-induced oxidative stress and hepatotoxicity in Swiss albino mice. Indian J. Exp. Biol..

[123] Sarkar R., Hazra B., Mandal N. (2012). Reducing power and iron chelating property of *Terminalia chebula* (Retz.) alleviates iron induced liver toxicity in mice. BMC Complement. Altern. Med..

[124] Zhang Y., Li H., Zhao Y., Gao Z. (2006). Dietary supplementation of baicalin and quercetin attenuates iron overload induced mouse liver injury. Eur. J. Pharmacol..

[125] Estuningtyas A., Wahyuni T., Wahidiyat P.A., Poerwaningsih E.H., Freisleben H. (2019). Mangiferin and mangiferin-containing leaf extract from *Mangifera foetida* L. for therapeutic attenuation of experimentally induced iron overload in a rat model. J. Herbmed Pharmacol..

[126] Baldwin C., Olarewaju O. (2020). Hemolytic anemia. StatPearls.

[127] Phillips J., Henderson A.C. (2018). Hemolytic anemia: Evaluation and differential diagnosis. Am. Fam. Phys..

[128] Avula V.V., Bora D., Upreti S., Jonathan S.K., Rijal S. (2015). Effect of aqueous extract of *Brassica oleraceae* var italica (Broccoli) inflorescence in phenylhydrazine induced anemic rats. Bull. Pharm. Res..

[129] Bavhure B., Borive M., Kadima J. (2014). Haematic and hepatroprotective potentials of *Hypoestes triflora* aqueous leaf extract in guinea-pigs. Int. J. Pharm. Sci. Res..

[130] Muhammad A.A., Ibrahim R.B., Aminu C., Abbas A.Y., Kabiru A., Bisallah C.I. (2020). Activity of aqueous extract of *Phyllanthus niruri* Linn in 2,4-dinitrophenylhydrazine induced anaemic rats. J. Pharm. Pharmacol. Res..

[131] Midala B.P., Teru P.A., Umaru H.A. (2017). Effect of *Gossypium hirsutum* L. ethanol leaf extract on phenylhydrazine-induced anaemic rats. J. Nat. Sci. Res..

[132] Goorani S., Koohi M., Zangeneh A., Zangeneh M., Moradi R. (2018). Pharmacological evaluation of anti-anemic property of aqueous extracts of *Falcaria vulgaris* leaf in rats. Comp. Clin. Path..

[133] Gheith I., El-Mahmoudy A. (2018). Laboratory evidence for the hematopoietic potential of *Beta vulgaris* leaf and stalk extract in a phenylhydrazine model of anemia. Braz. J. Med. Biol. Res..

[134] Nadro M.S., Modibbo A.A. (2014). Effects of *Pterocarpus erinaceus* stem bark aqueous extract on anemic rats. Sci. Res. J..

[135] Osafanme I.L., Duniya S.V., Chukwuemeka N.A.P., Mercy O., Adejoh I.P. (2019). Haematinic effects of aqueous extract of *Lophira lanceolata* leaves in phenylhydrazine-induced anaemia in wistar rats. Asian J. Res. Biochem..

[136] Sani H.L., Malami I., Hassan S.W., Alhassan A.M., Halilu M.E., Muhammad A. (2015). Effects of standardized stem bark extract of *Mangifera indica* L. in wistar rats with 2,4-dinitrophenylhydrazine-induced haemolytic anaemia. Pharmacogn. J..

[137] Adebayo M.A., Enitan S.S., Owonikoko W.M., Igogo E., Ajeigbe K.O. (2017). Haematinic properties of methanolic stem bark and fruit extracts of *Ficus sur* in rats pre-exposed to phenylhydrazine-induced haemolytic anaemia. Afr. J. Biomed. Res..

[138] Onyeabo C., Achi N.K., Ekeleme-Egedigwe C.A., Ebere C.U., Okoro C.K. (2017). Haematological and biochemical studies on *Justicia carnea* leaves extract in phenylhydrazine induced-anemia in albino rats. Acta Sci. Pol. Technol. Aliment..

[139] Iwalewa E.O., Omisore N., Daniyan O., Adewunmi C., Taiwo B.J., Fatokun O.A., Oluborode I.O. (2009). Elemental compositions and anti-anemic property of *Harungana madagascariensis* stem bark. Bangladesh J. Pharmacol..

[140] Sènou M., Tchogou A.P., Dougnon T.V., Agossadou A., Assogba F., Kinsiclounon E.G., Koudokpon H., Fah L., Fanou B., Akpovi D.C. (2016). Efficiency of *Sorghum bicolor* extract in the treatment of induced anemia on Wistar rats. Int. J. Biosci..

[141] Oladele J.O., Oyeleke O.M., Awosanya O., Olowookere B.D., Oladele O.T. (2020). Fluted Pumpkin (*Telfairia occidentalis*) protects against phenyl hydrazine-induced anaemia and associated toxicities in rats. Adv. Tradit. Med..

[142] Luka C.D., Abdulkarim M., Adoga G.I., Tijjani H., Olatunde A. (2014). Anti-anaemic potential of aqueous extract of *Spinacia oleracea* leaf in phenylhydrazine-treated rats. N. Y. Sci. J..

[143] Akah P.A., Okolo C.E., Ezike A.C. (2009). The haematinic activity of the methanol leaf extract of *Brillantasia nitens* Lindau (Acanthaceae) in rats. Afr. J. Biotechnol..

[144] Sarkiyayi S., Abubakar M.A. (2018). Effect of ethanol leaf extract of *Acacia nilotica* on phenyl hydrazine induced anemia in rats. IOSR J. Pharm. Biol. Sci..

[145] Aduwamai U.H., Abimbola M.M., Ahmed Z.H. (2018). Effect of *Solanum nigrum* methanol leaf extract on phenylhydrazine induced anemia in rats. Jordan J. Biol. Sci..

[146] Pandey S., Ganeshpurkar A., Bansal D., Dubey N. (2016). Hematopoietic effect of *Amaranthus cruentus* extract on phenylhydrazine-induced toxicity in rats. J. Diet. Suppl..

[147] Ogbe R.J., Adoga G.I., Abu A.H. (2010). Antianaemic potentials of some plant extracts on phenyl hydrazine-induced anaemia in rabbits. J. Med. Plants Res..

[148] Ologundudu A., Ologundudu A., Ololade I.A., Obi F.O. (2009). Effect of *Hibiscus sabdariffa* anthocyanins on 2,4-dinitrophenylhydrazine-induced hematotoxicity in rabbits. Afr. J. Biochem. Res..

[149] Yamoah A., Adosraku R., Amenu J., Baah M.K., Abaye D.A. (2020). Evaluation of the haematinic activities of extracts of *Justicia secunda* Vahl leaves in red blood cells of laboratory rats. J. Biosci. Med..

[150] Khan I., Shaikh H. (2020). Cooley anemia. StatPearls.

[151] Mariani R., Trombini P., Pozzi M., Piperno A. (2009). Iron metabolism in thalassemia and sickle cell disease. Mediterr. J. Hematol. Infect. Dis..

[152] Fung E.B., Harmatz P., Milet M., Ballas S.K., De Castro L., Hagar W., Owen W., Olivieri N., Smith-Whitley K., Darbari D. (2007). Multi-Center Study of Iron Overload Research Group. Morbidity and mortality in chronically transfused subjects with thalassemia and sickle cell disease: A report from the multi-center study of iron overload. Am. J. Hematol..

[153] Svobodová A., Walterová D., Psotová J. (2006). Influence of silymarin and its flavonolignans on H2O2-induced oxidative stress in human keratinocytes and mouse fibroblasts. Burns.

[154] Alidoost F., Gharagozloo M., Bagherpour B., Jafarian A., Sajjadi S.E., Hourfar H., Moayedi B. (2006). Effects of silymarin on the proliferation and glutathione levels of peripheral blood mononuclear cells from beta-thalassemia major patients. Int. Immunopharmacol..

[155] Cunningham-Rundles S., Giardina P.J., Grady R.W., Califano C., McKenzie P., De Sousa M. (2000). Effect of transfusional iron overload on immune response. J. Infect. Dis..

[156] Ezer Ü., Gülderen F., Çulha V.K., Akgül N., Gürbüz Ö. (2002). Immunological status of Thalassemia syndrome. Pediatr. Hematol. Oncol. J..

[157] Loizzo M.R., Tundis R., Menichini F., Pugliese A., Bonesi M., Solimene U., Menichini F. (2010). Chelating, antioxidant and hypoglycaemic potential of *Muscari comosum* (L.) Mill. bulb extracts. Int. J. Food Sci. Nutr..

[158] Bitis L., Kultur S., Melikoglu G., Ozsoy N., Can A. (2010). Flavonoids and antioxidant activity of *Rosa agrestis* leaves. Nat. Prod. Res..

[159] Ebrahimzadeh M.A., Nabavi S.M., Nabavi S.F. (2009). Correlation between the in vitro iron chelating activity and poly phenol and flavonoid contents of some medicinal plants. Pak. J. Biol. Sci..

[160] Mandel S., Weinreb O., Reznichenko L., Kalfon L., Amit T. (2006). Green tea catechins as brain-permeable, non toxic iron chelators to “iron out iron” from the brain. J. Neural. Transm. Suppl..

[161] Jomova K., Valko M. (2011). Importance of iron chelation in free radical-induced oxidative stress and human disease. Curr. Pharm. Des..

[162] Hatcher H.C., Singh R.N., Torti F.M., Torti S.V. (2009). Synthetic and natural iron chelators: Therapeutic potential and clinical use. Future Med. Chem..

[163] Gazak R., Walterova D., Kren V. (2007). Silybin and silymarin—New and emerging applications in medicine. Curr. Med. Chem..

[164] Darvishi Khezri H., Salehifar E., Kosaryan M., Aliasgharian A., Jalali H., Hadian Amree A. (2016). Potential Effects of Silymarin and Its Flavonolignan Components in Patients with β-Thalassemia Major: A Comprehensive Review in 2015. Adv. Pharmacol. Sci..

[165] Gharagozloo M., Karimi M., Amirghofran Z. (2013). Immunomodulatory effects of silymarin in patients with β-thalassemia major. Int. Immunopharmacol..

[166] Moayedi B., Gharagozloo M., Esmaeil N., Maracy M.R., Hoorfar H., Jalaeikar M. (2013). A randomized double-blind, placebo-controlled study of therapeutic effects of silymarin in β-thalassemia major patients receiving desferrioxamine. Eur. J. Haematol..

[167] Hagag A., Elfaragy M., Elrifaey S., Abd El-Lateef A. (2015). Therapeutic value of combined therapy with Deferiprone and Silymarin as iron chelators in Egyptian Children with Beta Thalassemia major. Infect. Disord. Drug Targets.

[168] Hagag A.A., Elfrargy M.S., Gazar R.A., El-Lateef A.E.A. (2013). Therapeutic value of combined therapy with deferasirox and silymarin on iron overload in children with beta thalassemia. Mediterr. J. Hematol. Infect. Dis..

[169] Franco S.S., De Falco L., Ghaffari S., Brugnara C., Sinclair D.A., Matte’ A., Iolascon A., Mohandas N., Bertoldi M., An X. (2014). Resveratrol accelerates erythroid maturation by activation of FoxO3 and ameliorates anemia in beta-thalassemic mice. Haematologica.

[170] Kalpravidh R.W., Siritanaratkul N., Insain P., Charoensakdi R., Panichkul N., Hatairaktham S., Srichairatanakool S., Phisalaphong C., Rachmilewitz E., Fucharoen S. (2010). Improvement in oxidative stress and antioxidant parameters in beta-thalassemia/Hb E patients treated with curcuminoids. Clin. Biochem..

[171] Srichairatanakool S., Thephinlap C., Phisalaphong C., Porter J.B., Fucharoen S. (2007). Curcumin contributes to in vitro removal of non-transferrin bound iron by deferiprone and desferrioxamine in thalassemic plasma. Med. Chem..

[172] Amer J., Goldfarb A., Rachmilewitz E.A., Fibach E. (2008). Fermented papaya preparation as redox regulator in blood cells of beta-thalassemic mice and patients. Phytother. Res..

[173] Koonyosying P., Kongkarnka S., Uthaipibull C., Svasti S., Fucharoen S., Srichaviratanakoo S. (2018). Green tea extract modulates oxidative tissue injury in beta-thalassemic mice by chelation of redox iron and inhibition of lipid peroxidation. Biomed. Pharmacother..

[174] Thephinlap C., Phisalaphong C., Lailerd N., Chattipakorn N., Winichagoon P., Vadolas J., Fucharoen S., Porter J.B., Srichairatanakool S. (2011). Reversal of cardiac iron loading and dysfunction in thalassemic mice by curcuminoids. Med. Chem..

[175] Thephinlap C., Ounjaijean S., Khansuwan U., Fucharoen S., Porter J.B., Srichairatanakool S. (2007). Epigallocatechin-3-gallate and epicatechin-3-gallate from green tea decrease plasma non-transferrin bound iron and erythrocyte oxidative stress. Med. Chem..

[176] Koonyosying P., Tantiworawit A., Hantrakool S., Utama-ang N., Cresswell M., Fucharoen S., Porter J., Srichairatanakool S. (2020). Consumption of a green tea extract-curcumin drink decreases blood urea nitrogen and redox iron in β-thalassemia patients. Food Funct..

[177] Argyropoulou M.I., Astrakas L. (2007). MRI evaluation of tissue iron burden in patients with bea-thalassaemia major. Pediatr. Radiol..

[178] Cao A., Galanello R. (2010). Beta-thalassemia. Genet. Med..

[179] Nemeth E. (2010). Hepcidin in beta-thalassemia. Ann. N. Y. Acad. Sci..

[180] Gharagozloo M., Moayedi B., Zakerinia M., Hamidi M., Karimi M., Maracy M., Amirghofran Z. (2009). Combined therapy of silymarin and desferrioxamine in patients with beta-thalassemia major: A randomized double-blind clinical trial. Fundam. Clin. Pharmacol..

[181] El-Shanshory M., Hablas N., Aboonq M., Ragab A., Attia M., Keshk W., Mariah R., Baghdadi H., Ayat M., Zolaly M. (2019). *Nigella sativa* improves anemia, enhances immunity and relieves iron overload-induced oxidative stress as a novel promising treatment in children having beta-thalassemia major. J. Herb. Med..

[182] Fibach E., Tan E.S., Jamuar S., Ng I., Amer J., Rachmilewitz E.A. (2010). Amelioration of oxidative stress in red blood cells from patients with beta-thalassemia major and intermedia and E-beta-thalassemia following administration of a fermented papaya preparation. Phytother. Res..

[183] Mohammadi E., Tamaddoni A., Qujeq D., Nasseri E., Zayeri F., Zand H., Gholami M., Mir S.M. (2018). An investigation of the effects of curcumin on iron overload, hepcidin level, and liver function in β-thalassemia major patients: A double-blind randomized controlled clinical trial. Phytother. Res..

[184] Upanan S., Pangjit K., Uthaipibull C., Fucharoen S., Mckie A., Srichairatanakool S. (2015). Combined treatment of 3-hydroxypyridine-4-one derivatives and green tea extract to induce hepcidin expression in iron-overloaded β-thalassemic mice. Asian Pac. J. Trop. Biomed..

[185] Marks P.W., Lazarus H.M., Schmaier A.H. (2019). Anemia: Clinical approach. Concise Guide to Hematology.

[186] Forget B.G., Bunn H.F. (2013). Classification of the disorders of hemoglobin. Cold Spring Harb. Perspect. Med..

[187] Imaga N.A. (2013). Phytomedicines and nutraceuticals: Alternative therapeutics for sickle cell anemia. Sci. World J..

[188] Ameh S.J., Tarfa F.D., Ebeshi B.U. (2012). Traditional Herbal Management of Sickle Cell Anemia: Lessons from Nigeria. Anemia.

[189] Dash B., Archana Y., Satapathy N., Naik S. (2013). Search for antisickling agents from plants. Pharmacogn. Rev..

[190] Mpiana P.T., Tshibangu D.S.T., Shetonde O.M., Ngbolua K.N. (2007). In vitro antidrepanocytary actvity (anti-sickle cell anemia) of some congolese plants. Phytomedicine.

[191] Oniyangi O., Cohall D.H. (2015). Phytomedicines (medicines derived from plants) for sickle cell disease. Cochrane Database Syst. Rev..

[192] Abraham D.J., Mehanna A.S., Wireko F.C., Whitney J., Thomas R.P., Orringer E.P. (1991). Vanillin, a potential agent for the treatment of sickle cell anemia. Blood.

[193] Silva D.G.H., Belini E., de Almeida E.A., Bonini-Domingos C.R. (2013). Oxidative stress in sickle cell disease: An overview of erythrocyte redox metabolism and current antioxidant therapeutic strategies. Free Radic. Biol. Med..

[194] Al Balushi H., Hannemann A., Rees D., Brewin J., Gibson J.S. (2019). The effect of antioxidants on the properties of red blood cells from patients with sickle cell anemia. Front. Physiol..

[195] Cristina Marcarini J., Ferreira Tsuboy M.S., Cabral Luiz R., Regina Ribeiro L., Beatriz Hoffmann-Campo C., Segio Mantovani M. (2011). Investigation of cytotoxic, apoptosis-inducing, genotoxic and protective effects of the flavonoid rutin in HTC hepatic cells. Exp. Toxicol. Pathol..

[196] Yang J., Guo J., Yuan J. (2008). In vitro antioxidant properties of rutin. LWT Food Sci. Technol..

[197] Nafees S., Rashid S., Ali N., Hasan S.K., Sultana S. (2015). Rutin ameliorates cyclophosphamide induced oxidative stress and inflammation in Wistar rats: Role of NFκB/MAPK pathway. Chem. Biol. Interact..

[198] Guo R., Wei P., Liu W. (2007). Combined antioxidant effects of rutin and vitamin C in Triton X-100 micelles. J. Pharm. Biomed. Anal..

[199] Muhammad A., Waziri A.D., Forcados G.E., Sanusi B., Sani H., Malami I., Abubakar I.B., Oluwatoyin H.Y., Adinoyi O.A., Mohammed H.A. (2019). Sickling-preventive effects of rutin is associated with modulation of deoxygenated haemoglobin, 2,3-bisphosphoglycerate mutase, redox status and alteration of functional chemistry in sickle erythrocytes. Heliyon.

[200] Muhammad A., Waziri A.D., Forcados G.E., Sanusi B., Sani H., Malami I., Abubakar I.B., Muhammad A., Muhammad R.A., Mohammed H.A. (2020). Sickling-suppressive effects of chrysin may be associated with sequestration of deoxy-haemoglobin, 2,3-bisphosphoglycerate mutase, alteration of redox homeostasis and functional chemistry of sickle erythrocytes. Hum. Exp. Toxicol..

[201] Mozar A., Charlot K., Sandor B., Rabaï M., Lemonne N., Billaud M., Hardy-Dessources M.D., Beltan E., Pandey R.C., Connes P. (2015). Pfaffia paniculata extract improves red blood cell deformability in sickle cell patients. Clin. Hemorheol. Microcirc..

[202] Onyegeme-Okerenta B.M., Essien E.B., Esin J.I. (2019). Anti-sickling properties of aqueous extracts of *Dennettia tripetala* and *Physalis angulata*. Asian J. Biol. Sci..

[203] Mgbemene C.N., Ohiri F.C. (1999). Anti-sickling potential of *Terminalia catappa* leaf extract. Pharm. Biol..

[204] Moody J.O., Ojo O.O., Omotade O.O., Adeyemo A.A., Olumese P.E., Ogundipe O.O. (2003). Anti-sickling potential of a Nigerian herbal formula (ajawaron HF) and the major plant component (*Cissus populnea* L. CPK). Phytother. Res..

[205] Imaga N., Gbenle G.O., Okochi V., Akanbi S., Edeoghon S.O., Oigbochie V., Kehinde M., Bamiro S. (2009). Antisickling property of *Carica papaya* leaf extract. Afr. J. Biochem. Res..

[206] Imaga N., Gbenle G.O., Okochi V., Adenekan S., Edeoghon S.O., Kehinde M., Bamiro S., Ajiboye A., Obinna A. (2010). Antisickling and toxicological profiles of leaf and stem of *Parquetina nigrescens* L.. J. Med. Plants Res..

[207] Naiho A.O., Okonkwor B.C., Okoukwu C. (2015). Anti-sickling and membrane stabilizing effects of *Carica papaya* leaf extract. Br. J. Med. Med. Res..

[208] Ouattara B., Jansen O., Angenot L., Guissou I.P., Frédérich M., Fondu P., Tits M. (2009). Antisickling properties of divanilloylquinic acids isolated from *Fagara zanthoxyloides* Lam. (Rutaceae). Phytomedicine.

[209] Elekwa I., Monanu M.O., Anosike E.O. (2005). Effects of aqueous extracts of *Zanthoxylum macrophylla* roots on membrane stability of human erythrocytes of different genotypes. Biokemistri.

[210] Elekwa I., Monanu M.O., Anosike E.O. (2005). In vitro effects of aqueous extracts of *Zanthoxylum macrophylla* roots on adenosine triphosphatases from human erythrocytes of different genotypes. Biokemistri.

[211] Atabo S., Umar I.A., James D.B., Mamman A.I. (2016). Sickled erythrocytes reversal and membrane stabilizing compounds in *Telfairia occidentalis*. Scientifica.

[212] Ogwutum F.E., Uwakwe A., Wegwu M.O., Osuoha J.O. (2018). In vitro investigation of the anti-sickling and erythrocyte membrane stabilizing potentials of *Elaesis guineensis* Jacq Flower. Int. J. Biochem. Biophys..

[213] Anosike C.A., Igboegwu O.N., Nwodo O.F.C. (2018). Antioxidant properties and membrane stabilization effects of methanol extract of *Mucuna pruriens* leaves on normal and sickle erythrocytes. J. Tradit. Complement. Med..

[214] Pauline N., Cabral B.N., Anatole P.C., Jocelyne A.M., Bruno M., Jeanne N.Y. (2013). The in vitro antisickling and antioxidant effects of aqueous extracts *Zanthoxyllum heitzii* on sickle cell disorder. BMC Complement. Altern. Med..

[215] Nwaoguikpe R.N., Ujowundu C.O., Okwu G.N. (2013). The antisickling potentials of four Curcubits (*T. occidentalis*, *C. maxima*; *C. sativus* and *C. lonatus*). Sch. J. App. Med. Sci..

[216] Afolabi I.S., Osikoya I.O., Fajimi O.D., Usoro P.I., Ogunleye D.O., Bisi-Adeniyi T., Adeyemi A.O., Adekeye B.T. (2012). Solenostemon monostachyus, *Ipomoea involucrata* and *Carica papaya* seed oil versus Glutathione, or *Vernonia amygdalina*: Methanolic extracts of novel plants for the management of sickle cell anemia disease. BMC Complement. Altern. Med..

[217] Onyegeme-Okerenta B.M., Essien E.B. (2019). In vitro antisickling potentials of ethanol extract of *Annona muricata*, *Delonix regia* and *Senna alata*. Recent Trends Pharm. Sci. Res..

[218] Tshilanda D.D., Onyamboko D.N., Babady-Bila P., Ngbolua K.T., Tshibangu D.S., Dibwe E.D.F., Mpiana P.T. (2015). Anti-sickling activity of ursolic acid isolated from the leaves of *Ocimum gratissimum* L. (Lamiaceae). Nat. Prod. Bioprospect..

[219] Adejumo O.E., Kolapo A.L., Folarin A.O. (2012). *Moringa oleifera* Lam. (Moringaceae) grown in Nigeria: In vitro antisickling activity on deoxygenated erythrocyte cells. J. Pharm. Bioallied. Sci..

[220] Abere T.A., Okoye C.J., Agoreyo F.O., Eze G.I., Jesuorobo R.I., Egharevba C.O., Aimator P.O. (2015). Antisickling and toxicological evaluation of the leaves of *Scoparia dulcis* Linn (Scrophulariaceae). BMC Complement. Altern. Med..

[221] Oduola T., Adeniyi F., Ogunyemi E., Bello I., Idowu T. (2006). Antisickling agent in an extract of unripe pawpaw (*Carica papaya*): Is it real?. Afr. J. Biotechnol..

[222] Ibrahim H., Sani F.S., Danladi B.H., Ahmadu A.A. (2007). Phytochemical and antisickling studies of the leaves of *Hymenocardia acida* Tul (Euphorbiaceae). Pak. J. Biol. Sci..

[223] Nwaoguikpe R.N., Braide W., Ezejiofor T.I. (2010). The effect of Aloe vera plant (*Aloe barbadensis*) extracts on sickle cell blood (HbSS). Afri. J. Food Sci. Tech..

[224] Takasu J., Uykimpang R., Sunga M.A., Amagase H., Niihara Y. (2006). Aged garlic extract is a potential therapy for sickle-cell anemia. J. Nutr..

[225] Omer R.H., Yousif N.A., Fadlalla T.A., Elradi W.E.O., Elbasheir M.M., allah E.I.A. (2020). In vitro antisickling activity of *Moringa oleifera* extracts on sickle cells. Res. Sq..

[226] Biapa P.C.N., Yembeau N.L., Chetcha B., Fotsing C.K., Nkwikeu P.J.N., Telefo P.B., Oben J.E., Pieme C.A. (2019). The anti-sickling mechanism of *Amphimas pterocarpoides* (Cesalpiniaceae) against sickle cell anemia. Acad. J. Med. Plants.

[227] Syed M.M., Doshi P.J., Dhavale D.D., Doshi J.B., Kate S.L., Kulkarni G., Sharma N., Uppuladinne M., Sonavane U., Joshi R. (2019). Potential of isoquercitrin as antisickling agent: A multi-spectroscopic, thermophoresis and molecular modeling approach. J. Biomol. Struct. Dyn..

[228] Fadlalla T.A., Yousif N.A., Omer R.H., Elbasheir M.M., Elradi W.O., Abdallah E.I. (2020). In vitro anti sickling activity of *Hyphaene thebaica* fruits extract on sickle cell. Res. Sq..

[229] Syed M.M., Doshi P.J., Bharshankh A., Dhavale D.D., Kate S.L., Kulkarni G., Doshi J.B., Kulkarni M.V. (2020). Repurposing of genistein as anti-sickling agent: Elucidation by multi spectroscopic, thermophoresis, and molecular modeling techniques. J. Biomol. Struct. Dyn..

[230] Ikechukwu E.L., Okafor P.N., Egba S.I. (2020). In vitro assessment of the anti-sickling properties of *Buchholzia coriacea* and *Mucuna pruriens* seed extracts. In Vitro Cell. Dev. Biol. Anim..

[231] Segel G.B., Lichtman M.A., Kaushansky K., Williams W.J. (2010). Aplastic anemia: Acquired and inherited. Williams Hematology.

[232] Scheinberg P., Chen J. (2013). Aplastic anemia: What have we learned from animal models and from the clinic. Semin. Hematol..

[233] Young N.S., Calado R.T., Scheinberg P. (2006). Current concepts in the pathophysiology and treatment of aplastic anemia. Blood.

[234] Ding S.X., Chen T., Wang T., Liu C., Lu W., Fu R. (2018). The risk of clonal evolution of granulocyte colony-stimulating factor for acquired aplastic anemia: A systematic review and meta-analysis. Acta Haematol..

[235] Georges G., Doney K., Storb R. (2018). Severe aplastic anemia: Allogeneic bone marrow transplantation as first-line treatment. Blood Adv..

[236] Schoettler M., Nathan D. (2018). The pathophysiology of acquired aplastic anemia: Current concepts revisited. Hematol. Oncol. Clin. N. Am..

[237] Yamazaki H. (2018). [Acquired aplastic anemia: Recent advances in pathophysiology and treatment]. [Rinsho Ketsueki] Jpn. J. Clin. Hematol..

[238] Moore C.A., Krishnan K. (2020). Aplastic Anemia. StatPearls.

[239] Scheinberg P. (2012). Aplastic anemia: Therapeutic updates in immunosuppression and transplantation. Hematol. Am. Soc. Hematol. Educ. Program.

[240] Young M.E., Potter V., Kulasekararaj A.G., Mufti G.J., Marsh J.C. (2013). Haematopoietic stem cell transplantation for acquired aplastic anaemia. Curr. Opin. Hematol..

[241] Passweg J.R., Aljurf M. (2013). Treatment and hematopoietic SCT in aplastic anemia. Bone Marrow Transplant..

[242] Gao R.L., Chong B.H. (2012). Research and development of the effective components of Panaxdiol saponin as new Chinese patent medicine for treating hemocytopenia. Chin. J. Integr. Med..

[243] Gao R.L., Chen X.H., Lin X.J., Qian X.D., Xu W.H., Chong B.H. (2007). Effects of notoginosides on proliferation and upregulation of GR nuclear transcription factor in hematopoietic cells. Acta Pharmacol. Sin..

[244] Yin L.M., Jiang H.F., Wang X., Qian X.D., Gao R.L., Lin X.J., Chen X.H., Wang L.C. (2013). Effects of sodium copper chlorophyllin on mesenchymal stem cell function in aplastic anemia mice. Chin. J. Integr. Med..

[245] Sun X., Gao R.L., Lin X.J., Xu W.H., Chen X.H. (2013). Panax notoginseng saponins induced up-regulation, phosphorylation and binding activity of MEK, ERK, AKT, PI-3K protein kinases and GATA transcription factors in hematopoietic cells. Chin. J. Integr. Med..

[246] Yin L.M., Wang X., Qian X.D., Lin X.J., Chen X.H., Gao R.L. (2012). Effects of Panax notoginseng saponins on proliferation and differentiation in NIH3T3 cells. Chin. J. Integr. Med..

[247] Zhao Y., Sun X., Yu X., Gao R., Yin L. (2018). Saponins from Panax notoginseng leaves improve the symptoms of aplastic anemia and aberrant immunity in mice. Biomed. Pharmacother..

[248] Dijiong W., Xiaowen W., Linlong X., Wenbin L., Huijin H., Baodong Y., Yuhong Z. (2019). Iron chelation effect of curcumin and baicalein on aplastic anemia mouse model with iron overload. Iran J. Basic Med. Sci..

[249] Zhang L., Feng H., He Y., Zhao J., Chen Y., Liu Y. (2017). Ginseng saponin Rb1 enhances hematopoietic function and dendritic cells differentiation. Acta Biochim. Biophys. Sin..

[250] Zheng Z.Y., Yu X.L., Dai T.Y., Yin L.M., Zhao Y.N., Xu M., Zhuang H.F., Chong B.H., Gao R.L. (2019). Panaxdiol saponins component promotes hematopoiesis and modulates T lymphocyte dysregulation in aplastic anemia model mice. Chin. J. Integr. Med..

[251] Wang Y., Yan T., Ma L., Liu B. (2015). Effects of the total saponins from *Dioscorea nipponica* on immunoregulation in aplastic anemia mice. Am. J. Chin. Med..

